# Synesthesia is linked to large and extensive differences in brain structure and function as determined by whole-brain biomarkers derived from the HCP (Human Connectome Project) cortical parcellation approach

**DOI:** 10.1093/cercor/bhae446

**Published:** 2024-11-15

**Authors:** Jamie Ward, Julia Simner, Ivor Simpson, Charlotte Rae, Magda del Rio, Jessica A Eccles, Chris Racey

**Affiliations:** School of Psychology and Sussex Neuroscience, University of Sussex, Brighton, BN1 9QH, United Kingdom; School of Psychology and Sussex Neuroscience, University of Sussex, Brighton, BN1 9QH, United Kingdom; School of Engineering and Informatics, University of Sussex, Brighton, BN1 9QH, United Kingdom; School of Psychology and Sussex Neuroscience, University of Sussex, Brighton, BN1 9QH, United Kingdom; School of Psychology and Sussex Neuroscience, University of Sussex, Brighton, BN1 9QH, United Kingdom; Department of Clinical Neuroscience, Brighton and Sussex Medical School (BSMS), Brighton, BN1 9QH, United Kingdom; Neurodevelopmental Service, Sussex Partnership NHS Foundation Trust, Worthing, BN13 3EP, United Kingdom; School of Psychology and Sussex Neuroscience, University of Sussex, Brighton, BN1 9QH, United Kingdom

**Keywords:** synesthesia/synaesthesia, biomarker, functional connectome, structural covariance, hyper-connectivity, development

## Abstract

There is considerable interest in understanding the developmental origins and health implications of individual differences in brain structure and function. In this pre-registered study we demonstrate that a hidden subgroup within the general population—people with synesthesia (e.g. who “hear” colors)—show a distinctive behavioral phenotype and wide-ranging differences in brain structure and function. We assess the performance of 13 different brain-based biomarkers (structural and functional MRI) for classifying synesthetes against general population samples, using machine learning models. The features in these models were derived from subject-specific parcellations of the cortex using the Human Connectome Project approach. All biomarkers performed above chance with intracortical myelin being a particularly strong predictor that has not been implicated in synesthesia before. Resting state data show widespread changes in the functional connectome (including less hub-based connectivity). These brain-based individual differences *within* the neurotypical population can be as large as those that differentiate neurotypical from clinical brain states.

## Introduction

People with synesthesia are a largely hidden subgroup within the general population: they appear outwardly unremarkable, but their inner worlds consist of extraordinary experiences of “merged sensations” such as the number “five” tasting of sour oranges or triggering a shiny blue color. Synesthetic experiences are not spontaneous but elicited (one stimulus triggers another), subjectively involuntary (lack of control over the synesthesia), and internally reliable over time. Synesthesia emerges in childhood (Simner and Bain 2013), if not sooner, and typically persists throughout life with a population prevalence of several percent (Simner et al. 2006). These unusual experiences are likely to be an outcome of an atypical neurodevelopmental trajectory that not only gives rise to synesthesia itself but also gives rise to a wider phenotype encompassing differences in cognition (e.g. heightened memory), personality (e.g. traits such as high openness to experience), and behavior (e.g. choices of occupation; Rich et al. 2005; Rouw and Scholte 2016; Ward et al. 2019). These phenotypic differences are typically small-to-medium in effect size. However, multiple smaller differences can equate to a statistically large difference between synesthetes and non-synesthetes (Ward and Filiz 2020). As such, synesthesia represents an important test case for understanding individual differences in brain and behavior—a topic that has generated much recent interest in an era of big data (Seghier and Price 2018; Greene et al. 2022). Here we show that brain-based biomarkers predict the presence of synesthesia at a similar level of accuracy as those developed for psychiatric and neurological conditions (Woo et al. 2017). In effect, individual differences *within* the general population may be just as large as those that distinguish between typical and clinical states. We also show across measures of personality, cognition, and clinical traits (e.g. anxiety) that synesthesia is linked to a behavioral profile that distinguishes them from non-synesthetes with large predictive accuracy.

The phenomenology of synesthesia—the fact that one stimulus elicits an unrelated sensation—has led to speculation that the brains of synesthetes are more connected (Hubbard and Ramachandran 2005; Bargary and Mitchell 2008) and/or less modular (Baron-Cohen et al. 1993). One influential theory has linked synesthesia to an early stage in normal development where brain regions are more connected and less specialized (Maurer and Mondloch 2006). This early phase may, to some degree, be retained by synesthetes but reversed in neurotypical development of everyone else (i.e. becoming less connected, more specialized). For example, during normal development the cortex becomes thinner as synapses are lost (Fjell et al. 2015) and certain white-matter pathways are scaled back (Falchier et al. 2002). In line with this, some studies have found areas of increased cortical gray matter density and white matter organization (fractional anisotropy) in adult synesthetes (Rouw and Scholte 2010). One specific claim is that increased connectivity may predominantly be between adjacent regions (Hubbard et al. 2011), perhaps due to changes in formation of cortical areal boundaries (Bargary and Mitchell 2008). For example, neuroanatomical proximity between regions specialized for color and text could explain why graphemes (letters and numbers) often elicit colors. Note that these accounts assume a common brain profile of what it is to be synesthetic, rather than having completely separate explanations for each type of synesthesia (grapheme–color, word-taste, etc.). Moreover, such changes are assumed not to be limited to the domain of synesthesia itself (e.g. generally more connectivity between adjacent regions in the model of Hubbard et al. 2011). This idea remains essentially untested in neuroimaging, but there is supportive evidence from other sources. Different types of synesthesia often co-occur both within individuals (Ward and Simner 2022) and within families (Barnett et al. 2008). The more types of synesthesia a person has, then the more extreme their behavioral phenotype (Ward and Filiz 2020) arguably because they are outcomes of a more extreme brain profile.

Although there are many neuroimaging studies of synesthesia (Rouw et al. 2011), there is a lack of consensus over the findings. Indeed, some researchers have concluded that its “signature in the brain may be out of reach with present brain imaging techniques” (Hupe and Dojat 2015). These discrepancies likely stem from differences in imaging methodologies (VBM, DTI, fMRI), different characteristics of the synesthetes themselves, and failures to replicate due to sample size inadequacy. There is also a key distinction to be made between previous mass univariate approaches, which require conservative thresholding (to minimize false positives) or that limit analyses to a priori regions of interest, versus multivariate machine learning approaches that consider many variables in parallel. Machine learning methods are powerful because multiple weaker effects, including those falling below a significance threshold in conventional approaches, may nonetheless constitute a reliable overall pattern: a brain-based biomarker. Machine learning methods are also robust due to the process of k-fold cross-validation in which participants are assigned to independent training sets (for developing the algorithm) and test sets (for measuring the accuracy of the predictions).

This study aimed to apply best practice in biomarker discovery (Woo et al. 2017) with state-of-the-art neuroimaging protocols from the Human Connectome Project, HCP (Van Essen et al. 2013; Harms et al. 2018). The core aspect of the HCP approach is the division of the cortex into distinct areas (termed parcellation) such that each individual area can be differentiated from adjacent areas on at least 1 of several dimensions such as their pattern of resting state BOLD connectivity to other regions or structural features such as myelination (Glasser et al. 2016). A unique map of 360 cortical regions is obtained for every individual and, in the present study, we take different sets of *n* = 360 variables (e.g. for surface area, myelination) to train a machine learning classifier to predict group status (synesthete versus control). No previous neuroimaging study of synesthesia has approached it in this way. For some biomarkers we compare inter-regional similarity/correlations between pairs of regions (effectively the upper triangle of a 360 × 360 array) noting that structural covariation has been interpreted as an indirect measure of connectivity, arising from coordinated neurodevelopment (Alexander-Bloch et al. 2013). A practical advantage is that the HCP protocols are designed to be interoperable, enabling comparison with other normative and special samples collected elsewhere with this method. Here we combine locally acquired datasets (synesthetes, controls) with publicly available normative samples from HCP projects termed Young Adults, HCP YA (Harms et al. 2018) and adults from the HCP Development/Aging (D/A) dataset (Harms et al. 2018), using standard harmonization procedures to control for site effects (Horng et al. 2022).

To pre-empt our findings, we find that all the brain-based biomarkers considered here can classify synesthetes from controls above chance, typically with large effect sizes. The brain-based biomarkers are found across synesthetes irrespective of the number of types a person has or their projector–associator status (i.e. a contrast between synesthetes, depending on whether their synesthetic colors are perceived as being external in space, or internal to the mind’s eye). For the brain-based biomarkers the variables within each biomarker correspond to different regions extracted using the HCP parcellation procedure. Thus, for example, we don’t have biomarkers that combine completely different variables (e.g. surface area and thickness). This is for ease of interpretation. There is no mathematical reason why different kinds of variables can’t be in the same predictive model and, indeed, this is the case for our behavioral analysis (e.g. this includes questionnaire scores, accuracy data, etc.).

## Method

The methods and analysis were pre-registered (https://osf.io/ycqgd/) and additions and changes are explicitly noted in Appendix 1. This project has generated novel datasets that are openly available, and described in detail elsewhere (Racey et al. 2023), but no analyses of the dataset have been previously reported. In addition, me make use of normative samples acquired and analyzed using the HCP protocol.

### Participants

A total of 237 participants completed all of the behavioral assessments. This included 128 people with synesthesia (mean age = 36.01, SD = 13.42; gender 96:25:4 for female:male:other) and 109 participants classed as non-synesthetes (mean age = 34.59, SD = 13.11; gender 78:29:2 for female:male:other). Synesthetes were recruited from a database of volunteers held by our research group, and controls were recruited opportunistically (matching the groups for age and gender). Although it was not part of our recruitment/inclusion strategy, we note that synesthetes had achieved somewhat lower levels of formal education (27% to age 18, 46% to undergraduate, 27% to postgraduate) than the equivalent controls (15% to age 18, 45% to undergraduate, 40% to postgraduate). Within this wider sample, the neuroimaging session was completed by *n* = 102 synesthetes (mean age = 35.38, SD = 13.20; gender 78:23:1 for female:male:other) and *n* = 25 non-synesthetes (mean age = 35.08, SD = 14.14; gender 19:6:0 for female:male:other). All synesthetes had at least 1 verified type, with details of the types and verification procedure outlined in the related Data Descriptor paper (Racey et al. 2023). The types of synesthesia reported were as follows, noting that many individuals have several types: language-color (*n* = 100), sequence-space (*n* = 94), visualized sensations (*n* = 69), personification (*n* = 47), hearing-motion (*n* = 39), tickertape (*n* = 37), mirror-touch (*n* = 32), language-taste (*n* = 17), other smell/taste concurrents (*n* = 11), and language-touch (*n* = 10). Participants gave informed consent for both participation and data sharing and the project was approved by the Brighton and Sussex Medical School (BSMS) Research Governance and Ethics Committee.

Additional controls to the *n* = 25 noted above, for the neuroimaging analysis, were recruited from 3 sources. In this way, our four sets of controls form a 2 × 2 design that contrasts differences in the scanner location (Sussex versus HCP consortium) and minor differences in MRI sequence (HCP YA versus HCP D/A). Our main motivation was to utilize the larger external datasets of the HCP consortium (giving higher statistical power), while providing sufficient assurance that between-group differences could not be better explained by differences in site, scanner, or imaging protocol. We accessed a group of demographically matched healthy volunteers (*n* = 25, 6 males, mean age = 35.96, SD = 12.71) scanned at University of Sussex on unrelated projects that used the HCP YA protocol. We also downloaded publicly available fully processed data from the HCP YA (Van Essen et al. 2013) and HCP D/A (Harms et al. 2018) datasets, selecting *n* = 300 participants from each, and matching to synesthetes by sex at birth (70 and 73 males) and to be as similar as possible in terms of mean age (30.18 and 36.48, SDs of 3.75 and 13.97, respectively; noting that there is not a uniform coverage of ages in these databases). For the HCP YA, pairs of twins were never included but there were a small number of non-twin sibling pairs (*n* = 33). Thus, overall, we had *n* = 650 controls and *n* = 102 synesthetes for creating brain-based biomarkers.

For the behavioral data, an a priori power analysis was performed using G^*^Power based on 2-tailed independent *t*-tests with alpha = 0.05. At a power of 0.8, an N of 100 (per group) is sufficient for detecting a Cohen’s d of 0.40 and above. For brain-based biomarkers these are hard to estimate a priori (due to their multivariate nature), but there is no evidence that sample sizes beyond 100 are linked to improved classification accuracy (Woo et al. 2017). Instead, classification accuracy depends on factors such as the ability to measure the trait in the first place, and the sophistication of the neuroimaging and classification protocols.

### Procedure: behavioral assessments

All participants completed both sessions of the study (clinical measures and cognitive measures) on the online platform Qualtrics (Provo, UT). They were given separate links for the different sessions and asked to complete them both within a week (although the timing between sessions is not critical). The order of the measures within each session were fixed and the order is as listed in Table 1. The choice of measures was largely motivated by prior research showing group differences relating to these constructs (Rouw and Scholte 2016; Carmichael et al. 2019; Ward and Filiz 2020). Exceptions include depression (Carmichael et al. 2019, failed to find an effect), subclinical symptoms relating to PTSD (but for formal diagnosis after trauma, Hoffman et al. 2019), and hypermobility (which has been linked to other forms of neurodiversity; Csecs et al. 2022). The Data Descriptor paper (Racey et al. 2023) describes the administration and scoring of these measures including quality assurance metrics (e.g. Cronbach’s alpha).

**Table 1:** An overview of the behavioral measures. Measures are listed in the order in which they were taken (over 2 sessions). The number of items is shown together with the number of dependent variables, DVs, for each measure (i.e. composites of items or some other item-derived scale). + For a 2-factor GSQ proposal, see Sapey-Triomphe et al. (2018).

Online session 1: clinical measures and synesthesia types					
Construct	Measure	Type	Items	DVs	Reference
Synesthesia	A mixture of bespoke questions and Projector–Associator questionnaire	Questionnaire	7–25	N/A	Rouw & Scholte (Rouw and Scholte 2007)
Autistic traits	AQ Autism Spectrum Quotient	Questionnaire	50	5	Baron-Cohen et al. (2001)
Sensory sensitivity	GSQ Glasgow Sensory Questionnaire	Questionnaire	42	1+	Robertson and Simmons (2013).
Anxiety	ASI-3 Anxiety Sensitivity Index	Questionnaire	18	3	Taylor et al. (2007)
Depression	DASS-21 Depression, Anxiety, and Stress Scale	Questionnaire	21	3	Henry and Crawford (2005)
PTSD	Impact of Event Scale—Revised	Questionnaire	22	3	Maercker and Schutzwohl (1998)
Hypermobility	5PQ, Five-part questionnaire	Questionnaire	5	1	Glans et al. (2020)
Session 2: cognitive and personality measures					
Construct	Measure	Type	Items	DVs	Reference
Personality	BFI-2 Big Five Inventory	Questionnaire	60	5	Soto and John (2017)
Memory (learning phase)	24 words letter judgment	Test	24	N/A	Ward and Filiz (2020)
Creativity	Alternate Uses	Test	6	2	Guilford et al. (1978)
Memory (test phase)	48 words old/new judgment with confidence rating	Test	48	4	Ward and Filiz (2020)
Imagery	PSI-Q Plymouth Sensory Imagery Questionnaire	Questionnaire	35	7	Andrade et al. (2014)
Intelligence	Ravens Matrices	Test	11	1	Bors and Stokes (1998)

### Procedure: neuroimaging

The procedure for acquiring the MRI data for the synesthetes and locally sourced controls, together with pre-processing and quality control metrics, are described in detail by Racey et al. (Racey et al. 2023). In brief, we used multimodal MRI scans acquired using the HCP D/A protocol obtained from the Connectome Coordination Facility. The scanner hardware mirrored that being used at the imaging centers currently acquiring the HCP D/A dataset and consisted of a Siemens 3 T Prisma scanner with a 32-channel head coil with multiband echo-planar imaging, EPI, sequences. The cortical surface is divided into 360 parcellated areas (180 in each hemisphere) using a fully automatic, multi-modal processing pipeline. The parcellations are derived from a combination of T1-weighted (T1w), T2-weighted (T2w), and resting-state functional MRI (rfMRI; Glasser et al. 2013). Control data obtained from the HCP D/A dataset (see Participants section) had been collected and analyzed in identical ways.

Two further control groups used the slightly different HCP YA acquisition procedures (Van Essen et al. 2012). Data is reported to be very similar when the same participants are scanned twice using HCP YA and D/A acquisition procedures (Harms et al. 2018). The HCP YA dataset (Van Essen et al. 2012) had 58 min of resting state (compared to 26 min for D/A), 2 structural scans (instead of 1 for D/A) and 0.8 mm isotropic voxels (instead of 0.7 mm for D/A). The HCP YA protocol run at University of Sussex for 2 previous studies (used for *n* = 25 control participants) was also obtained via the Connectome Coordination Facility and used the 3 T sequences described in Van Essen et al. (2012) but reduced the amount of resting state data collected (from 58 to 46 min) and the number of T1w and T2w scans (from 2 to 1).

Harmonization across sites was achieved using the ComBat procedure, which uses Empirical Bayes to eliminate differences across samples (across sites, scanners, or batches) while preserving variables of interest (Horng et al. 2022). This process was applied to: sub-cortical volumes and eTIV; parcellated intracortical myelin, thickness, and surface area; and resting state partial correlations (noting that all other biomarkers are derived from these). The harmonization model also included information about batch (the four sources of data), the age and sex of the participant, and group status (synesthete, control).

### Analyses: behavioral

Overall, there were 35 dependent variables. When these variables are factors within a scale they were analyzed as analysis of variance, but otherwise as *t*-tests [applying false discovery rate (FDR) for multiple comparisons]. Bayes factors were computed using the room-to-move heuristic (Dienes 2019). We also used machine learning (a Random Forest classifier) to predict group status (synesthete versus control) across the entire range of variables, using 10-fold cross validation and 1 tuned hyper-parameter (the number of variables in each tree varying between 3 and 10). Random Forests are based on the concept of classification using decision trees, but with the extension of using multiple decision trees (here the default of *n* = 500) each presented with different subsets of variables. It has the advantage of being able to handle many variables (e.g. N variables > > N participants) and is robust against over-fitting. Each decision tree performs a classification (in effect a “vote” for either synesthete or control) and votes are pooled across trees. Area under the curve (AUC) is the main variable for describing the overall performance of the model (with 1 being perfect and 0.5 being chance) across all possible thresholds (i.e. vote share). Specificity and sensitivity are also noted; corresponding, respectively, to the proportion of synesthetes classed as synesthetes and the proportion of controls classed as controls. It is also possible to get a continuous metric for each participant (proportion of votes predicting synesthesia or control) which is indicative of how easy or hard it was to classify that person (a 50/50 vote share indicating they cannot be easily classified).

### Analyses: neuroimaging

The neuroimaging analyses had 2 main forms: a more standard mass univariate approach (although noting that this is done at a parcel rather than voxel level) and a multivariate machine learning approach (for biomarker discovery and validation). Independent *t*-tests were performed for each region with FDR applied to correct for multiple comparisons. The Random Forest machine learning algorithm, described above, was used with 5-fold cross-validation and down-sampling due to an imbalance in participant numbers (having considerably more controls than synesthetes). There was iterative refinement and testing of the algorithm over 3 steps termed discovery, demonstration, and generalization (Woo et al. 2017). We deliberately included synesthetes with multiple types (3+) in the earlier discovery phase as we expected this to be linked to larger effects. The discovery sample was used to set the optimal hyper-parameter (number of variables considered by each tree), and then this was applied to an otherwise comparable “demonstration” sample (i.e. having the same profile of 3+ types of synesthesia), and the “generalization” sample (with a looser criterion of 1+ types). The synesthetes were divided 25/25/52 across these samples (according to the number of types and randomly thereafter). Controls from the HCP YA and HCP D/A control datasets were randomly halved between discovery and demonstration samples, and the locally acquired control datasets were used in the discovery sample alone. However, we show that nothing of substance hinges on these decisions: single-step biomarkers based on including all 102 synesthetes and 650 controls yielded comparable performance (using 5-fold cross-classification and down-sampling). We establish chance performance baselines by shuffling the 102 + 650 group labels and re-running the algorithm over 1,000 permutations. The most important variables that drive classification were determined using the R package, VSURF (Genuer et al. 2015), which has been shown to perform favorably compared to other solutions (Speiser et al. 2019)—these are reported in Appendix 3. Appendix 6 contains an exploratory (not pre-registered) analysis of the degree of association between different biomarkers.

The biomarkers are derived from the subject-specific measures across the *n* = 360 cortical regions, or the second-order differences across these regions (i.e. derived from an *n* = 360 x 360 matrix comparing each region against every other). There were 2 exceptions to this: a biomarker based on subcortical and volumetric data (34 regions), and a biomarker for global surface area and estimated total intra-cranial volume (4 variables with the addition of sex and age). All biomarkers (aside from the subcortical volumes) were pre-registered and motivated by prior literature from synesthesia and/or neurodevelopmental individual-differences. These are summarized in Table 2.

**Table 2:** A summary of the thirteen biomarkers indicating the number of variables, how calculated, and rationale for including (in terms of prior research or theories).

Biomarker	Variables	Rationale
Surface area and eTIV	*n* = 4. Surface area (mm^2^), eTIV (estimated total intracranial volume, mm^3^), sex at birth, age.	Increased global SA reported in synesthetes after controlling for intracranial volume (Janke et al. 2009).
Regional surface area	*n* = 360. Calculated as % of total. Summed surface area of vertices within each region (weighted by cortical thickness).	Regional increases in SA reported in one prior study of synesthesia (Janke et al. 2009).
		
Volumetric measures of sub-cortex and other structures	*n* = 34. Extracted volumes (mm^3^) computed by FreeSurfer and rescaled to % of eTIV. The regions are: [gray matter] left and right amygdala, hippocampus, thalamus, ventral dienchephalon, caudate, cerebellum cortex, pallidum, putamen, accumbens area; [white matter] optic chiasm, five regions of corpus callosum (anterior, central, mid-anterior, mid-posterior, posterior), left and right cerebellum white matter; CSF, third ventricle, fourth ventricle, left and right lateral ventricles, and inferior lateral ventricles.	Differences in subcortical gray and white matter in synesthesia observed with VBM (Rouw and Scholte 2010; Simner et al. 2015)
Cortical thickness	*n* = 360. Unweighted, and regional estimates based on median rather than mean (see Raamana and Strother 2020).	Some prior evidence of increased thickness in some regions (Janke et al. 2009) and localized increases in gray matter density in VBM linked to synesthesia (Rouw and Scholte 2010)
Inter-regional differences in cortical thickness (pairwise)	*n* = (360 × 359)/2 followed by data reduction with PCA. Absolute difference in thickness between every pair of regions.	Based on the assumption that inter-connected regions develop a similar structural profile (Lerch et al. 2006). Cortical regions in synesthetes may be more similar to each other as a result of more inter-connectivity during development (Hänggi et al. 2011).
Inter-regional differences in cortical thickness (summed)	*n* = 360. Sum of differences calculated from above.	An aggregated measure of similarity of one region to all others, which one study showed to be linked to synesthesia (Hänggi et al. 2011).
Intra-cortical myelination	*n* = 360. Weighted version based on the 32 k resolution data.	No directly relevant prior research although other studies have noted inter-cortical white-matter differences (Simner et al. 2015)
Inter-regional differences in myelination (pairwise)	*n* = (360 × 359)/2 followed by data reduction with PCA. Absolute difference in myelination between every pair of regions.	Based on the assumption that strong connectivity during development results in a similar myelin profile (Ma and Zhang 2017). No relevant prior data from synesthesia.
Inter-regional differences in myelination (summed)	*n* = 360. Sum of differences calculated from above.	A possible indicator of coordinated development of regions as a proxy measure of connectivity. No relevant prior data from synesthesia.
Functional connectivity	*n* = (360 × 359)/2 followed by data reduction with PCA. Correlations (Pearson’s r) in the BOLD time series transformed into partial correlations (based on Menon and Krishnamurthy 2019).	Menon and Krishnamurthy (2019) report this method has better test–retest reliability and group discrimination (of sex) than others. Increased functional connectivity has been reported in synesthetes with resting state (Dovern et al. 2012) and task-based fMRI (Tomson et al. 2013).
Functional connectivity (centrality)	*n* = 360. Sum of all partial correlations (ignoring sign) from each region.	Higher values indicate that this region exerts stronger influence over other regions (i.e. acting like a hub).
Functional gradients (principal gradient)	*n* = 360. Gradient score for each region after applying PCA to functional connectome (set of Pearson’s correlations), using 1^st^ principal gradient (from visual cortices to association cortex).	Gradients are a form of data reduction in which regions are ordered according to the similarity of their resting state connectivity profile. No prior research on functional gradients relating to synesthesia, but differences reported for autism (Hong et al. 2019) and sensory sensitivity (Del Río et al. 2022).
Functional gradients (second gradient)	*n* = 360. Gradient score for each region after applying PCA to functional connectome, using second principal gradient. From somatomotor cortices to the endpoints of the first gradient (visual and association cortices). See Fig. 5.	As above.

## Results

### Behavioral

**Fig. 1:**
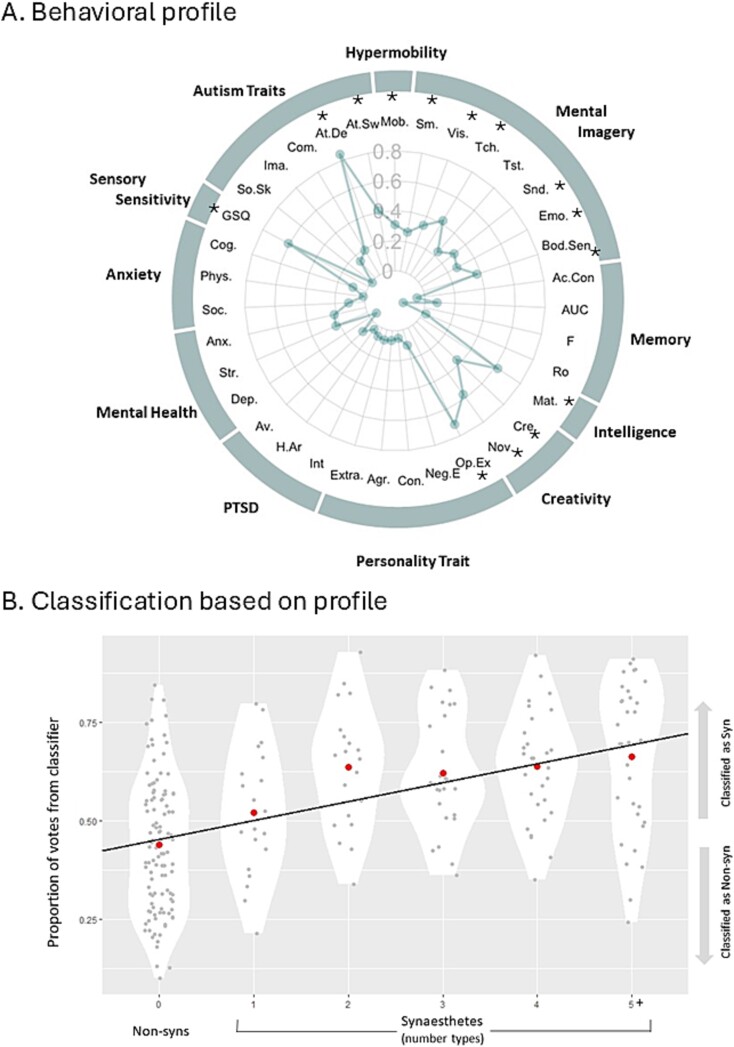
A) Synesthetic behavioral profile compared to controls showing effect sizes (Cohen’s d, synesthetes against controls) on 35 dependent variables. All effect sizes d > 0.26 are significant at *P* < 0.05 (uncorrected).B) Classification probabilities for individual participants organized by the number of types of synesthesia that they have. The large dots show the group means. A Random Forest classifier sets up a number of “learners” (decision trees) during the training phase, and each learner then casts a vote on each case in the test set (with the number of votes tallied). Thus, deviations away from 0.5 represents individuals who are easier to classify. People with 1 type of synesthesia are the hardest to classify, and those with 5+ are the easiest to classify. Abbreviations from clockwise: Mob. = hypermobility score; [imagery for] Sm. = smell, Vis. = vision, Tch. = touch, Tst. = taste, Snd. = sounds, Emo. = emotions, Bod.Sen = bodily sensations; [memory] Ac.Con = accuracy-confidence correlation, AUC = area-under-curve accuracy, F = familiarity (fitted), Ro = recollection (fitted), Mat. = Raven’s matrices; [creativity] Cre. = creativity score, Nov. = novelty score; [personality] Op.Ex = openness to experience, Neg.E = negative emotions, Con. = conscientiousness, Agr. = agreeableness, Extra. = extraversion; [PTSD] Int. = intrusions, H.Ar = hyperarousal, Ac. = avoidance; [mental health] Dep. = depression, Str. = stress, Anx. = anxiety; [anxiety] Soc. = social, Phys. = physical, Cog. = cognitive; GSQ = glasgow sensory questionnaire; [autism] So.Sk = social skills, Ima. = imagination, Com. = communication, At.De = attention-to-detail, At.Sw = attention switching.

Figure 1 shows the breakdown of effect sizes for each dependent variable comparing 128 synesthetes and 109 non-synesthetes (Appendix 4 contains descriptive statistics, *t*-tests with FDR corrections, and Bayes factors).

Multivariate approaches consider all variables in parallel (avoiding correction for multiple comparisons) essentially treating group differences as a form of pattern recognition. A machine learning classifier, using all 35 dependent variables, was able to predict group status with AUC = 0.806 (sensitivity = 0.782, specificity = 0.634), which is significantly better than chance [Χ^2^(1) = 43.522, *P* < 0.001]. The overall behavioral effect size was large as indicated both by the classifier accuracy (AUC = 0.806 corresponds to a large Cohen’s d of 1.221) and a multivariate equivalent of Cohen’s d (Mahalanobis D = 1.235, applying bias correction for small samples). Ease of classification was related to the number of types of synesthesia a person reports (r = 0.276, *n* = 128, *P* = 0.002) as shown in Fig. 1B. People with more types of synesthesia have a more distinctive profile that makes them easier to classify. Whether this association is truly linear or a step function (e.g. between 1 and 2 types) is unclear.

Considering univariate linear models, for the Autism Spectrum Quotient (AQ) there were significant main effects of group [F(1,235) = 11.559, *P* = 0.001, η^2^ = 0.047] and a significant interaction [F(4,940) = 9.544, *P* < 0.001, η^2^ = 0.039], with post hoc *t*-tests showing synesthetes reported good attention-to-detail (t(235) = 6.471, *P* < 0.001) and poor attention switching [t(235) = 3.202, *P* = 0.002]. For the anxiety sensitivity index (ASI-3) there was neither a significant effect of group [F(1,235) = 0.438, *P* = 0.509, η^2^ = 0.002] nor interaction [F(2,470) = 0.414, *P* = 0.661, η^2^ = 0.002], and similarly for the Impact of Events Scale [IES-R, group: F(1,235) = 0.316, *P* = 0.574, η^2^ = 0.001, interaction: F(2,470) = 0.305, *P* = 0.737, η^2^ = 0.001]. Sensory sensitivity (using the GSQ) was also significantly higher among synesthetes [t(235) = 4.732, *P* < 0.001]. Hypermobility (generalized joint laxity) was significantly higher in synesthetes [t(235) = 2.387, *P* = 0.018], although note that it did not survive FDR correction when applied to all 35 dependent variables. For the Depression, Anxiety and Stress Scale (DASS-21) there was no main effect of group [F(1,235) = 1.138, *P* = 0.287, η^2^ = 0.005] but a significant group X subscale interaction [F(2,470) = 3.887, *P* = 0.021, η^2^ = 0.016]. None of the 3 subscales showed significant group differences but the pattern was for numerically higher stress and anxiety in synesthetes alongside numerically lower depression. Taking available published clinical cut-offs where they were available (i.e. binarizing the continuous data), DASS Stress yielded a significant group differences [Χ^2^(1) = 4.130, *P* = 0.042], as did overall AQ [Χ^2^(1) = 8.516, *P* = 0.004], and joint hypermobility [Χ^2^(1) = 5.943, *P* = 0.015] but not for any other clinical measure (these are reported in full in Appendix 4).

For personality (BFI-2), there was a main effect of group [F(1,235) = 12.188, *P* = 0.001, η^2^ = 0.049] and interaction [F(4,940) = 3.090, *P* = 0.015, η^2^ = 0.013]. The latter was driven by significantly higher scores for synesthetes on Openness to Experience [t(235) = 5.458, *P* < 0.001]. For mental imagery, there was a significant main effect of group [F(1,235) = 8.869, *P* = 0.003, η^2^ = 0.036] but no interaction [F(61,410) = 0.428, *P* = 0.861, η^2^ = 0.02]. Synesthetes report more vivid mental imagery across multiple senses. On cognitive tests, synesthetes scored significantly higher on the matrix test of intelligence [t(235) = 4.752, *P* < 0.001]. For creativity, synesthetes scored significantly higher on the Alternate Uses Test, blind-scored judgments of both creativity [t(235) = 2.846, *P* = 0.005] and novelty [t(235) = 4.486, *P* < 0.001]. There were no significant differences relating to the memory measures, in contrast to prior research (the Bayes factor for memory familiarity was insensitive so we cannot conclude it is a null result).

Considering individual differences within synesthetes, these were explored in terms of number of types and, for grapheme–color synesthetes, their projector–associator status (i.e. whether synesthetic colors are externally perceived). Appendix 4 shows a breakdown of the variables broken down by number of types of synesthesia (and correlations). Our pre-registered analysis contrasted many (4+) versus few (1–3) types and here we find four significant differences: GSQ [t(126) = 2.587, *P* = 0.011], Openness to Experience [t(126) = 2.501, *P* = 0.007], AUT Creativity [t(126) = 2.664, *P* = 0.004], and IES-R Hyper-arousal [t(126) = 2.066, *P* = 0.020]. In each case, those with more types had more extreme scores (but none survive FDR correction for multiple comparisons). For grapheme–color synesthetes, there was no significant differences between projectors and associators on the classification votes [t(86) = 0.659, *P* = 0.512]. Exploratory analyses showed significant differences relating to one measure (not surviving correction for multiple comparisons but flagged here as being important for future replication). Projectors reported significantly higher PTSD-like phenomenology compared to associators (who were similar to controls) on the IES-R. This was found for overall score [t(86) = 2.644, *P* = 0.010], for the avoidance subscale [t(86) = 2.122, *P* = 0.038; noting that the other subscales showed the same trend], and when binarized by clinical cut-off [X^2^(1) = 4.803, *P* = 0.028].

### Brain-based biomarkers

#### Overview

**Fig. 2:**
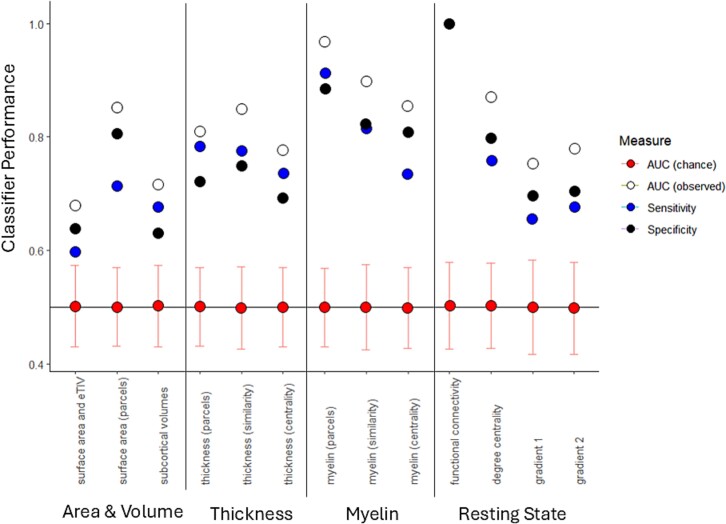
Classification performance for different biomarkers for held-out data (i.e. not part of the training set) in 5-fold validation using all participants with down-sampling for imbalanced groups. AUC = area under curve (chance performance is estimated by running the classifier with 1,000 sets of shuffled group labels and error bars show 95% confidence intervals); sensitivity = probability of classifying a synesthete as a synesthete; specificity = probability of classifying a control as a control.

The performance of the biomarkers, assessed in terms of group classification ability (AUC, sensitivity, specificity) are summarized in Fig. 2. All biomarkers performed significantly above chance, as assessed by repeatedly permuting the group labels. The best performing structural biomarkers related to intra-cortical myelin (these were also the best performing biomarkers overall), and the best performing functional biomarker was functional connectivity (estimated with partial correlations using the method of Menon and Krishnamurthy 2019). Both are novel results within the synesthesia literature. These biomarker results in themselves do not indicate the nature of the group differences (i.e. which regions are implicated and the direction of the effect). These are explored in detail below, following the order in which they are presented in Table 2 and Fig. 2.

#### Surface area

**Fig. 3:**
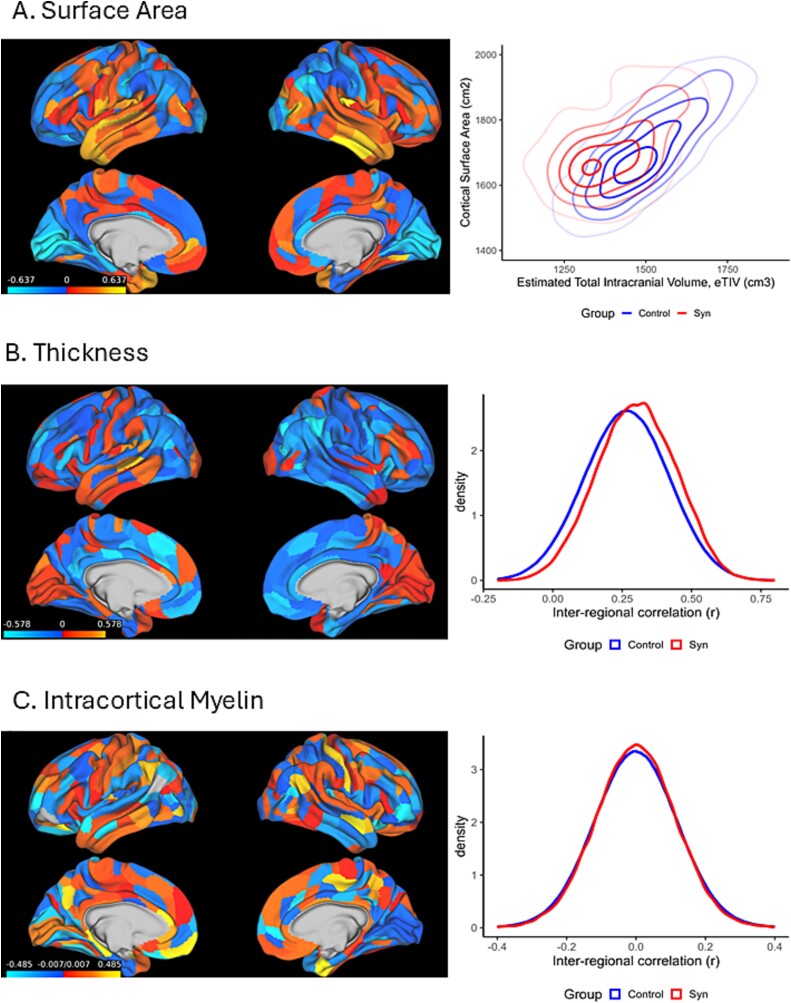
A) Cortical surface area, showing Cohen’s d regional differences calculated from group means as percentage of global area (left) and global differences in surface area and intracranial volume, eTIV (right). B) Cortical thickness group differences (left) and inter-regional similarity in cortical thickness shown here as a distribution of 360 × 360 correlations (r-values) between each cortical region for each group (visualized as mean of resamples of *n* = 102 controls). The corresponding biomarkers are constructed using a 360 × 360 set of difference scores in thickness (mm) between pairs of regions to permit subject-specific analyses (see Raamana and Strother 2020). C) Intracortical myelination group differences (left) and the pairwise inter-regional similarity correlations for myelin, calculated as described above (right). Brain images displayed on fsaverage whole-brain cortical surface reconstruction (top row = lateral view, bottom row = medial view) showing localized increases (red-yellow) or decreases (blue) in synesthetes relative to controls.

Figure 3A shows group-level differences in surface area broken down into the 360 cortical parcels together with overall surface area relative to volume (eTIV). Synesthetes showed a non-significant increase (16cm^2^) in global surface area [t(750) = 0.939, *P* = 0.348, d = 0.100], but significantly smaller intracranial volume, eTIVs [t(750) = 3.021, *P* = 0.003, d = −0.321] meaning that surface area per volume, cm^3^, is significantly higher [t(750) = 5.257, *P* < 0.001, d = 0.559]. This combination of surface area and eTIV is rare enough to act as a predictive biomarker for synesthesia (entering age and sex as additional variables into the classifier): the AUC was 0.680. Head size is largely determined by fusion of the skull plates in early childhood (Farkas et al. 1992) although eTIV, as measured by MRI based on inner skull surface, shows smaller changes through to adolescence (Mills et al. 2016).

At the regional level, there were 90 parcels with group differences in absolute area (mm^2^) that were significant after FDR correction, with most of these (*n* = 70) linked to cortical expansion but with some early visual regions showing reduced surface area (e.g. left and right V1, left V4). Expansions were most prominent in temporal lobe regions. When rescaled as percentage of total surface area, then a similar pattern is found (66 expansions and 46 reductions which all survive FDR correction). Regions of relative expansion and reduction tended not to be adjacent, that is increases in surface area likely arise from genuine expansions rather simply shifting boundaries between neighbors.

#### Subcortical and non-cortical volumes

The finding that eTIV tends to be smaller in synesthetes but cortical surface area is not scaled down proportionally (it is numerically larger than controls) led us to consider whether this applies to other non-cortical volumetric data (hence, not pre-registered). Indeed, there were 14 structures that were significantly enlarged as a percentage of eTIV in synesthetes compared to controls (after FDR correction)—see Table 3. No regions showed the reverse pattern. With regards to gray matter, synesthetes have proportionally larger (bilateral) cerebellar cortex, amygdalae, hippocampi, brainstem, (left) accumbens area, (right) caudate and ventral diencephalon (noting that, for the lateralized regions, similar effect sizes were found bilaterally but did not always meet the significance threshold). In term of white matter, synesthetes had higher volumes (relative to eTIV) in the cerebellum and mid corpus collosum.

**Table 3:** Volumetric data, expressed as percentage of eTIV (SD in parentheses), for structures showing significant differences between groups (FDR). Note that all listed structures are larger in synesthetes than controls. Non-significant results can be found in Appendix 7.

Region	Synesthetes	Controls	Cohen’s d, *P*-value (FDR)
Left cerebellum cortex	3.855 (0.449)	3.660 (0.414)	d = 0.466, *P* < 0.001
Right cerebellum cortex	3.897 (0.454)	3.689 (0.423)	d = 0.487, *P* < 0.001
Left cerebellum WM	0.954 (0.136)	0.895 (0.118)	d = 0.486, *P* < 0.001
Right cerebellum WM	0.923 (0.129)	0.874 (0.119)	d = 0.406, *P* = 0.002
Left amygdala	0.104 (0.016)	0.098 (0.012)	d = 0.485, *P* < 0.001
Right amygdala	0.110 (0.016)	0.102 (0.013)	d = 0.563, *P* < 0.001
Left hippocampus	0.278 (0.036)	0.268 (0.031)	d = 0.311, *P* = 0.009
Right hippocampus	0.284 (0.038)	0.271 (0.031)	d = 0.429, *P* = 0.003
Brainstem	1.478 (0.184)	1.395 (0.165)	d = 0.495, *P* < 0.001
Right ventral diencephalon	0.296 (0.037)	0.284 (0.031)	d = 0.378, *P* = 0.008
Left accumbens area	0.042 (0.009)	0.039 (0.008)	d = 0.429, *P* = 0.002
Right caudate	0.261 (0.033)	0.250 (0.032)	d = 0.331, *P* = 0.009
Corpus collosum (mid-anterior)	0.037 (0.008)	0.035 (0.008)	d = 0.321, *P* = 0.008
Corpus collosum (mid-posterior)	0.040 (0.008)	0.037 (0.008)	d = 0.289, *P* = 0.022

#### Thickness

For cortical thickness, there were 52 regions where the groups differed significantly following FDR (see Fig. 3B) with 5 regions showing thicker cortex in synesthetes and 47 regions showing thinner cortex. (Note that thickness does not scale with eTIV in the same way as surface area and we did not adjust for it; https://surfer.nmr.mgh.harvard.edu/fswiki/eTIV). Two additional biomarkers were derived from the parcellated thickness values based on a prior study. Hänggi et al. (2011) reported higher correlations in thickness between pairs of regions among synesthetes, and similarly when summing across all pairwise comparisons from a given region (equivalent to degree centrality in a network model). These are putative markers for greater coordination between brain regions during development, i.e. an indirect marker of connectivity (Lerch et al. 2006). Our results replicate that study insofar as synesthetes have higher r-values (when correlating thickness in region A against thickness in region B, etc.) than controls pooling across all pairwise comparisons. Moreover, we extend it to show that this pattern of inter-regional differences in thickness is sufficient to differentiate between groups in a machine learning classifier (as shown in Fig. 2), albeit at a similar AUC to the raw cortical thickness estimates themselves.

#### Intracortical myelination

Intracortical myelination has not been previously implicated in synesthesia either empirically or theoretically, but it serves as one of the best discriminating biomarkers that we considered. Taking a mass univariate approach, there were 70 regions where the groups differed significantly after correction for multiple comparisons with differences in both directions (32 regions where synesthesia is linked to more myelin, and 38 where it is linked to less). Group differences are shown in Fig. 3C and, unlike thickness and surface area differences, are a non-contiguous patchwork of differences. Two biomarkers are derived by contrasting myelin level in pairs of regions (15.0% or 9664/64620, paired differences are significant with FDR correction) and by contrasting absolute differences in myelin levels between each region and all others (10.3% or 37/360 are significant with FDR). To gain further insights we inter-regional correlations in myelination in each group, in the same way as that applied to thickness (Ma and Zhang 2017). In both groups, the set of correlations are numerically smaller and centered around zero. The presence of both positive and negative correlations is indicative of both competitive and cooperative inter-regional interactions in the establishment of myelination levels. The group differences in inter-regional correlations found for thickness (higher for synesthetes) did not extend to myelin.

#### Functional (resting state) connectomes

**Fig. 4:**
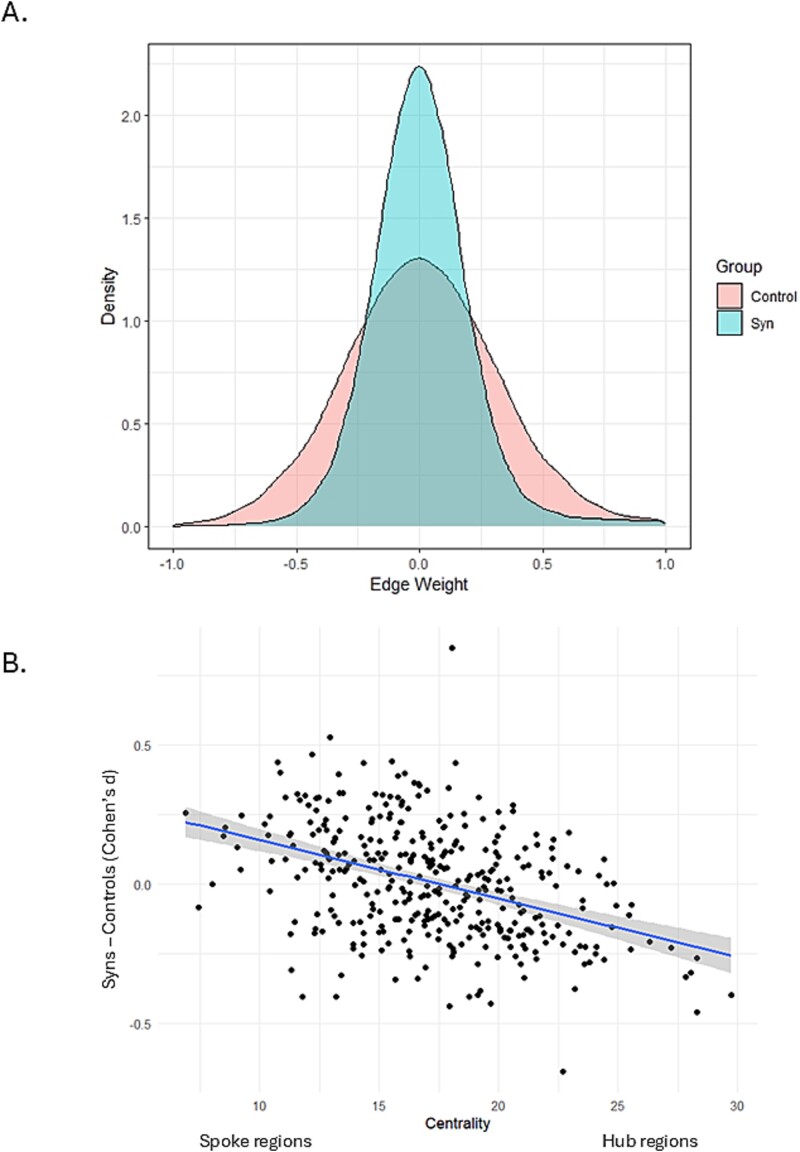
A) Distribution of partial correlations (edge weights) in the full functional connectome (upper triangle of 360 × 360 matrix) of synesthetes and controls. B) Summing the partial correlations at each region generates a measure of degree centrality (influence of that region over the network). For regions with high mean centrality (in controls), synesthetes have lower centrality than controls. For regions with low mean centrality (in controls), the pattern is reversed.

Correlations (Pearson’s r) in the BOLD timeseries between pairs of regions were calculated and these were then transformed into partial correlations, informed by prior research that the latter gives higher (test–retest) reliability than the former (Menon and Krishnamurthy 2019). Figure 4A shows the distribution of these partial correlations across the 2 groups, with controls showing more extreme correlations (in both directions). Of these 64,620 (= 360 × 359/2) connectivity values 14.6% show significant group differences (after FDR correction) and this constitutes our best performing biomarker. Performance of this biomarker remains very high when different harmonization steps are taken (by not providing the harmonization procedure with group labels), as described in Appendix 2.

The set of partial correlations were used to derive a measure relating to network structure. Degree centrality is the sum of the partial correlations (irrespective of sign) from a given region and this can be construed as the extent of its influence over the whole network. Synesthetes show significant differences (FDR correction) in 43 regions, with effects in both directions (25 positive, 18 negative). There is a structure to these differences insofar as more connected regions (hubs) are less central in synesthetes, and regions with lower centrality in controls are more connected in synesthetes (the correlation between centrality estimates in controls and size of group difference, d, is r = −0.409, *P* < 0.001 as shown in Fig. 4B). In effect, the functional connectome of synesthetes is “flatter” (less differentiating between hub and spoke).

**Fig. 5:**
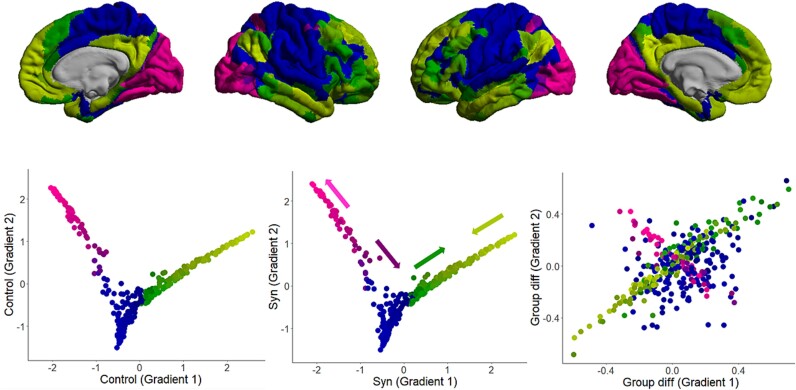
Gradient analyses of the functional connectome, where each point represents a different cortical parcel (*n* = 360) and color denotes eccentricity in the gradient (broadly: pink denotes visual regions, green is for “higher” association cortices, and blue is sensorimotor). Top: gradient scores projected on to lateral and medial surfaces of the brain (fsaverage). Bottom: gradient scores for controls (left) and synesthetes with arrows showing direction of group differences (middle), and group differences expressed as Cohen’s d (right). The latter shows that systematic group differences occur primarily within the “pink” and “green” axes.

A gradient analysis uses principal component analysis (PCA) to effectively “order” regions according to the similarity of connectivity profile and, like degree centrality, can be thought of as a form of data reduction. Here, we use the first 2 gradients as candidate biomarkers. The extracted gradients (Fig. 5) are consistent with previous research showing that the first gradient runs from visual cortices to association cortices (the default mode network being most extreme), and the second gradient runs from the sensorimotor cortices to the endpoints of the first gradient. Synesthetes and controls show an overall similarity in gradient structure with some group differences in gradient scores notably an “expansion” across visual regions (more dissimilarity between regions in synesthetes relative to controls) and “contraction” in associative cortex (more similarity between regions in synesthetes relative to controls). There were 50 and 46 regions (/360) where group differences were significant at FDR *P* < 0.05, across the first and second gradients respectively. Both gradients can act as reliable biomarkers of synesthesia (AUCs of 0.753 and 0.780 for gradients 1 and 2 respectively).

### Links between brain and behavior

Within synesthetes (*n* = 102), we tested whether classification accuracy from the biomarker (proportion of votes for each person from the Random Forest classifier) related to number of types of synesthesia or classification accuracy based on behavioral profile (as correlations), and, for grapheme–color synesthetes contrasting projectors and associators (via *t*-tests). None of these analyses yielded significant results (all p’s > 0.05). In summary, although we are able to distinguish between synesthetes and controls (using brain-based biomarkers) we were unable to link these differences to heterogeneity within the synesthetes. That is, we speculate that the brain-based biomarkers are characteristic of synesthesia, in its broadest sense, and this conclusion is also supported by the wide-ranging differences across the brain (as opposed to being localized to certain centers such as those relating to color).

## Discussion

This study has established that there are multiple brain-based biomarkers that distinguish between people with synesthesia and the general population. Although this makes it challenging to pinpoint specific causal mechanisms, the findings are consistent with the emerging view that neuroimaging phenotypes (e.g. relating to volume, thickness, area) are related to each other and to common latent constructs such as pleiotropic genetic effects (Warrier et al. 2023). By using machine learning, we establish that the biomarkers are reliable: that is, one can partition the dataset and show that an algorithm trained on one part of the dataset predicts group status in the independent held-out sample (using an area-under-curve, AUC, metric). For clinical diagnostics, an AUC > 0.8 is referred to as “good” and AUC > 0.9 is referred to as “excellent” (de Hond et al. 2022). Most of our biomarkers (and the behavioral phenotype) fall in the “good” range, with intra-cortical myelin and functional connectivity being “excellent.” These values are comparable to those reported for many neurological and psychiatric conditions (Woo et al. 2017; despite considerably more research effort going into them). But we make the important caveat that synesthesia is not a clinical disorder—it is a non-clinical form of neurodiversity and, hence, it is surprising that it is linked to such large effects. AUCs of 0.8 and 0.9 are equivalent to large Cohen’s d’s of 1.19 and 1.81 (Salgado 2018). While the constituent effect sizes in both behavior (Fig. 1) and brain differences (Fig. 3) tend to be in the small-to-medium range (Cohen’s d of 0.3–0.8), it is the reliable combination of multiple smaller differences that leads to overall large effects. This multivariate approach is one of the key differences between the present study and previous neuroimaging research on synesthesia which has relied on univariate (and mass univariate) analyses.

In terms of theoretical accounts of synesthesia, the most prominent theories center on the ideas of hyper-connectivity (broadly defined; Hubbard and Ramachandran 2005; Bargary and Mitchell 2008) and/or neurodevelopmental immaturity (based on the idea that synesthetes retain some of the characteristics of early brain development into adulthood; Maurer and Mondloch 2006). We discuss hyper-connectivity and possible development differences in turn.

In terms of (resting state) functional connectivity, synesthetes show differences in the pattern of connectivity but these are not well accounted for by a simple notion of hyper-connectivity. Summing the connectivity levels from each region (degree centrality) reveals that strongly connected regions in controls (hubs) have lower connectivity in synesthetes but non-hub regions show the reverse trend. Similarly, our gradient-based analysis also suggests differences in the organization of connectivity patterns, with regions organized along the principal gradient (from visual cortex to association cortex) showing both expansion and contraction (reflecting greater dissimilarity and similarity in the connectome, respectively). Aside from functional connectivity, greater inter-regional structural covariance has been taken as a marker of hyper-connectivity during development; that is, strong similarity between pairs of regions in terms of thickness or intracortical myelin content are taken as evidence of coordinated maturation of those regions (Alexander-Bloch et al. 2013). Here we find stronger correlations in synesthetes (relative to controls) for cortical thickness but not for myelin (see also Appendix 5 and a separate study by Hänggi et al. (2011)). These inter-regional relationships play out in a more extreme form in the synesthetes brain presumably by virtue of them being more inter-connected during development. However, the biological mechanisms (e.g. white matter tracts) that produce these coordinated patterns is not certain (Alexander-Bloch et al. 2013).

Although this study was not designed to directly test developmental questions, some speculations can be made. One hypothesis was that synesthetes might not undergo the same process of synaptic pruning from childhood to adulthood that normally results in cortical thinning (Fjell et al. 2015). We do not find the expected pattern: if anything the overall cortex of synesthetes is thinner rather than thicker (this could reflect changes in myelination rather than pruning; Whitaker et al. 2016). However, the increased inter-regional correlations in thickness in synesthesia is potentially indicative of a younger brain—this measure has been shown to decrease from 14 yr to adulthood (Vása et al. 2018). It would be important to replicate this using the HCP parcellation approach, and future research could apply machine leaning algorithms designed to predict the brain age to our synesthete sample to determine the extent to which they have a more youthful profile (Wood et al. 2022). The finding that speaks most closely to a different developmental trajectory is the observation that intracranial volume is smaller in synesthetes (noting that most variation in this measure is linked to the first 5 yr of life Farkas et al. 1992). Given that the brain itself is not scaled down as expected (synesthetes have normal cortical surface area and proportionally larger subcortical volumes), this pattern is consistent with delayed brain growth. That is, synesthetes may have had smaller brains when head growth stabilized (producing a reduced head size, a mild microcephaly) followed by later brain growth. This is a different pattern to that reported in autism (larger heads, normal-sized adult brains) which has been attributed to accelerated, rather than delayed, early brain growth (Redcay and Courchesne 2005). These different patterns are seemingly at odds with the observed co-morbidity of synesthesia and autism (van Leeuwen et al. 2020)—including the greater rate of autistic traits in the present synesthete sample. However, it could be explained by multiple subtypes of autism, including those relating to sex (macrocephaly is predominantly found in autistic males, Nordahl et al. 2011, and our sample is predominantly female).

A significant and original contribution of our biomarker approach has been to establish the importance of intracortical myelin in synesthesia. The functional role of intracortical myelin has been described as simplifying and stabilizing intracortical circuitry by, for example, limiting synaptic plasticity (Glasser et al. 2014). Synesthetes have been shown to have less perceptual narrowing (Maurer et al. 2020), which could be an outcome of this mechanism. Our results do not speak to differences in inter-cortical myelin, i.e. within the white matter, as studied by prior research in the field (Rouw and Scholte 2007; Simner et al. 2015). The omission of DTI (diffusion tensor imaging) represents a key limitation of our research. Note that DTI is not needed for the HCP parcellation pipeline, and our goal was to maximize sample size over breadth of neuroimaging modalities. Nevertheless, our biomarkers can act as a search tool for interrogating large neuroimaging datasets with a view to identifying individuals with a “synesthetic brain type” and unlocking data relating to DTI, health, genotype, etc. We would not need to test these individuals for synesthesia, as the biomarker itself would be considered a reasonable surrogate (Simner 2012). In effect, this would be a paradigm shift from studying synesthesia itself toward studying a distinctive form of neurodivergence that happens to be linked to synesthesia and, in reality, is also linked to a distinctive cognitive and behavioral profile (Ward 2019).

Another limitation is the possibility that the biomarker performance is an inflated estimate of the true group difference (aka over-fitting). Near-perfect classification performance is implausible given that some control samples were drawn from the general population and would not be solely comprised of non-synesthetes. The standard harmonization procedure used here (to account for differences across scanners) uses group labels to prevent genuine differences of interest being lost (Horng et al. 2022). We explored various alternative harmonization procedures in which group labels are not used: for example, when harmonizing to a reference sample comprising of an unlabelled mix of known synesthetes (minority) and known non-synesthetes (majority) that might be more representative of the general population. Performance drops but not precipitously (see Appendix 2). Moreover, our best performing biomarker has been shown in other research to have “excellent” discrimination within a non-clinical sample (Menon and Krishnamurthy 2019).

With regards to the observed behavioral profile, a limitation of the research is that we cannot discount the possibility that our sample of synesthetes are not representative of the population of synesthetes more generally. This could arise because synesthetes who volunteer for research have particular characteristics that distinguish them from other synesthetes (e.g. higher IQ) and from controls. However, previous research that tested large opportunistic samples have reported similar results to those here (e.g. Chun and Hupe, 2016). Future research should explore this more systematically. This could entail comparing synesthetes with other special samples, recruited in similar ways, to show that there are specific differences linked to being synesthetic. It would also be possible to use the brain-based biomarkers of synesthesia (or the behavioral equivalent) in cross-classification designs to determine whether they can predict other variables such as intelligence (high versus low IQ), likelihood of autism, etc.

Previous neuroimaging approaches have directly contrasted stimuli that do and do not induce synesthesia in fMRI (Rouw et al. 2011), with a view to understand the neural correlates of synesthesia. These studies have often highlighted the importance of particular regions such as V4 (specialized for color and form; Roe et al. 2012) and its interaction with parietal regions implicated in selection and attention. Although this kind of explanation of synesthesia remains viable, such differences occur against a backdrop of widespread brain changes and a distinctive phenotype that goes well beyond synesthesia itself. This includes higher mental imagery, attention to detail, sensory sensitivity, and better performance on measures of fluid intelligence (Raven’s matrices, creativity). However, the present research is not able to link specific brain changes to specific behaviors (e.g. because we don’t have behavioral measures from most of our controls). Prior research in synesthesia may have missed many of the findings reported in this study either because nobody looked for them (e.g. intracranial volume, intracortical myelin), because the sample sizes were under-powered, or because univariate voxel-based approaches are less suitable. Notably, the HCP approach acts as a principled form of data reduction (360 parcels instead of 100 K+ voxels) guided by individual anatomy. This may be an optimal level of granularity for biomarker discovery.

In terms of wider implications, there is significant interest in developing predictive models of brain-behavior relationships that go beyond a one-size-fits-all explanation and can accommodate individual differences (Seghier and Price 2018; Greene et al. 2022). Synesthesia is an important test case for this enterprise because synesthetes will be present in all other large normative datasets. They are not trivially rare (4 to 8% depending on the number and types included (Simner et al. 2006; Ward et al. 2018)) but nor are they common enough to override the central tendency. Thus, synesthetes are likely to appear as statistical noise within an averaged normative sample. This occurs despite the fact that there is a reliable brain and behavior phenotype and despite the fact that it represents a statistically large difference with respect to the residual neurotypical mean (the difference being a similar order of magnitude as that between the healthy mean and those of clinically diagnosed categories). Of course, there is no reason to believe that what we term neurotypical is divided solely into the binary categories of “synesthete” and “non-synesthete”. Instead, there may be many such groupings (varying greatly in prevalence) and many dimensions on which brain structure and function can differ. By chipping away at the idea of a neurotypical brain we may be left with the situation that nobody remaining is “normal” but, in doing so, we will hopefully lay the foundations for a better understanding the differences and similarities between people.

## Supplementary Material

Supplementary_Material_collated_harmonized_data_bhae446
