# Supplementary material for: Synesthesia is linked to large and extensive differences in brain structure and function as determined by whole-brain biomarkers derived from the HCP (Human Connectome Project) cortical parcellation approach
Source: Cereb Cortex. 2024 Nov 15;34(11):bhae446. doi: 10.1093/cercor/bhae446 (PMC11567774; doi:10.1093/cercor/bhae446)
Supplement: Supplementary_Material_collated_harmonized_data_bhae446 [file supplementary_material_collated_harmonized_data_bhae446.docx]

**Supplementary Material**

[**Appendix 1:** Divergence from Pre-registration](#Appendix1)

[**Appendix 2:** Interoperability Across Neuroimaging Datasets and Consistency of Results](#Appendix2)

[**Appendix 3:** Detailed Reporting of Biomarker Performance](#Appendix3)

[**Appendix 4:** Further Details of Behavioural Results](#Appendix4)

[**Appendix 5:** Inter-regional Correlations - Replication of Hänggi et al. (2011) and Extension to Intracortical Myelin](#Appendix5)

[Appendix 6: Associations between Biomarkers](#Appendix6)

[Appendix 7: Links to results files showing means and group differences by region](#Appendix7)

**Appendix 1: Divergence from Pre-registration**

The following tables summarise additions and changes with respect to the pre-registration.

*Tables S1 and S2. Additions and changes to the pre-registration, including a justification.*

| **Addition to pre-registered proposal** | **Rationale** |
| --- | --- |
| Sub-cortical and volumetric biomarker | These are automatically calculated by the analysis pipeline. The observed differences in cortical gray matter and eTIV prompted further investigation. |
| Analysing the clinically-related questionnaire data using published cut-offs. | The absence of significant results (when using continuous data) prompted further investigation. |
| No consideration of the problem of imbalanced classifiers [1] in the pre-registration. Addition of undersampling (aka down-sampling) when the number of controls exceeds the number of synaesthetes | If undersampling is not used then the classifier produces near perfect specificity but very poor sensitivity (i.e., classifying the whole sample as controls) |

| **Pre-registered Proposal** | **Change** | **Rationale** |
| --- | --- | --- |
| N = 400 participants downloaded from each of HCP YA and HCP D/A databases | N = 300 participants downloaded from each; 8 missing datasets for fMRI resting state (HCP D/A) | To achieve better matching by age and gender to the synaesthetes |
| No initial feature selection for large biomarkers (N = 64,620). All features entered into classifer. | Data reduction using PCA, and extracting all eigenvectors > 1 | Computationally challenging to implement. Generates too many noisy/redundant features. |
| Resting state data from all datasets. | No useable resting state data from the HCP YA database. | Downloadable data not available in concatenated ICA+FIX format. Multiple requests sent to HCP coordinators. |
| Leave-one-out cross-validation with AUC as model selection | Five-fold cross-validation with AUC as model selection | AUC cannot be calculated in leave-one-out |
| Control groups cannot be reliably discriminated from each other; more generally, any differences between syn v. control cannot be explained by differences in scanning protocol or scanner location. | Use of ComBat harmonization [2] across the four study batches | The pre-registration did not fully specify how this would be achieved. |
| Naive Bayes classifier which  acts as an aggregate classifier across biomarkers | Not conducted | Undersampling of the majority group (controls) prevents repeated measures from each classifier being obtained |

**Appendix 2: Interoperability Across Neuroimaging Datasets and Consistency of Results**

**Overall Biomarker Performance without Harmonization**

The analyses pipelines were also run on non-harmionized (raw) data, albeit with the exception of running the HCP YA dataset through Freesurfer v6.0 for volumetric and thickness data (to make it more comparable to the other samples all pre-processed in this way). Note that harmonization would also attempt to account for these differences albeit in a different way (based on the numerical distributions of the observed data).

Figure S1 shows the results of the overall biomarker performance for raw (non-harmonized) data. Whilst overall performance is generally similar there are significant differences in terms of the regional pattern of differences that drive classification, i.e. some differences reflect site differences and others reflect genuine group differences. Myelin remains a high performing biomarker both with and without harmonization. For functional connectivity, there are substantial differences according to whether harmonization is used (excellent classification) or not (worse classification). This reflect sizeable differences in the resting state data across sites.


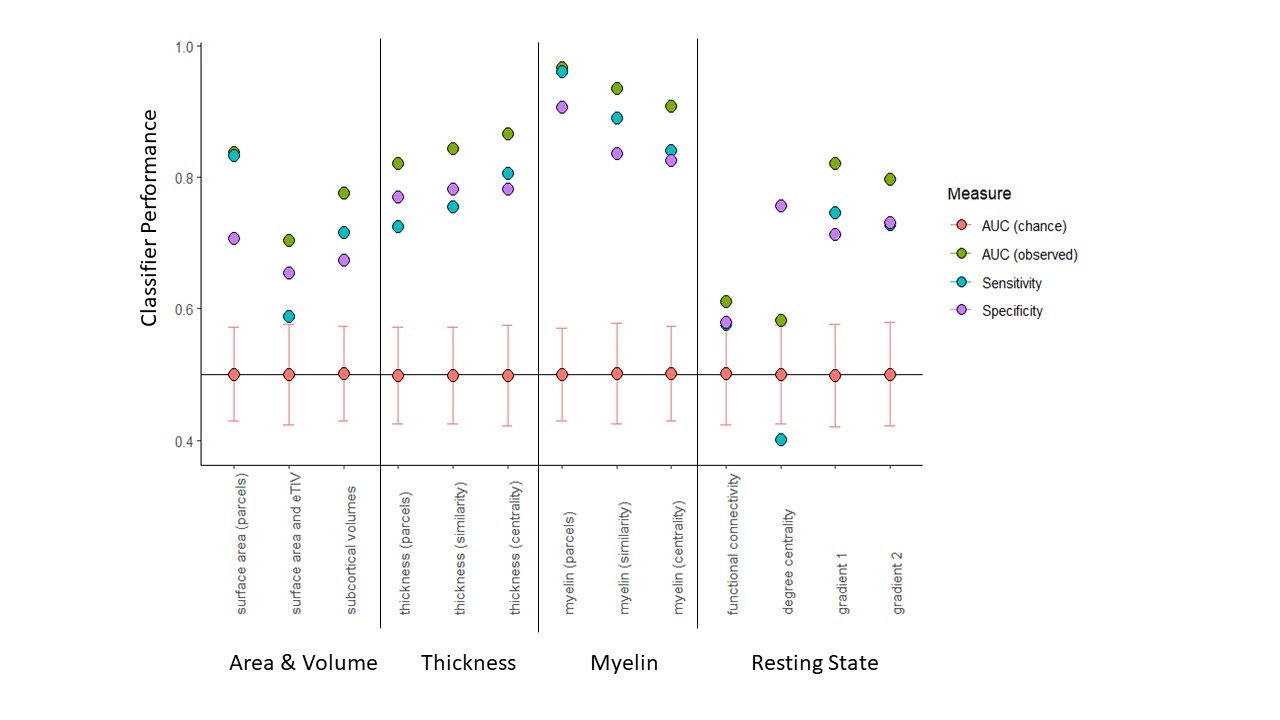


*Figure S1. Biomarker performance using raw data and following the pre-registered analysis plan (shown for all control and synaesthete data).*

Figure S2 shows the performance of the two sets of inter-regional correlations (myelin, thickness) are found across harmonization which acts on the size of the data but not the order.

**
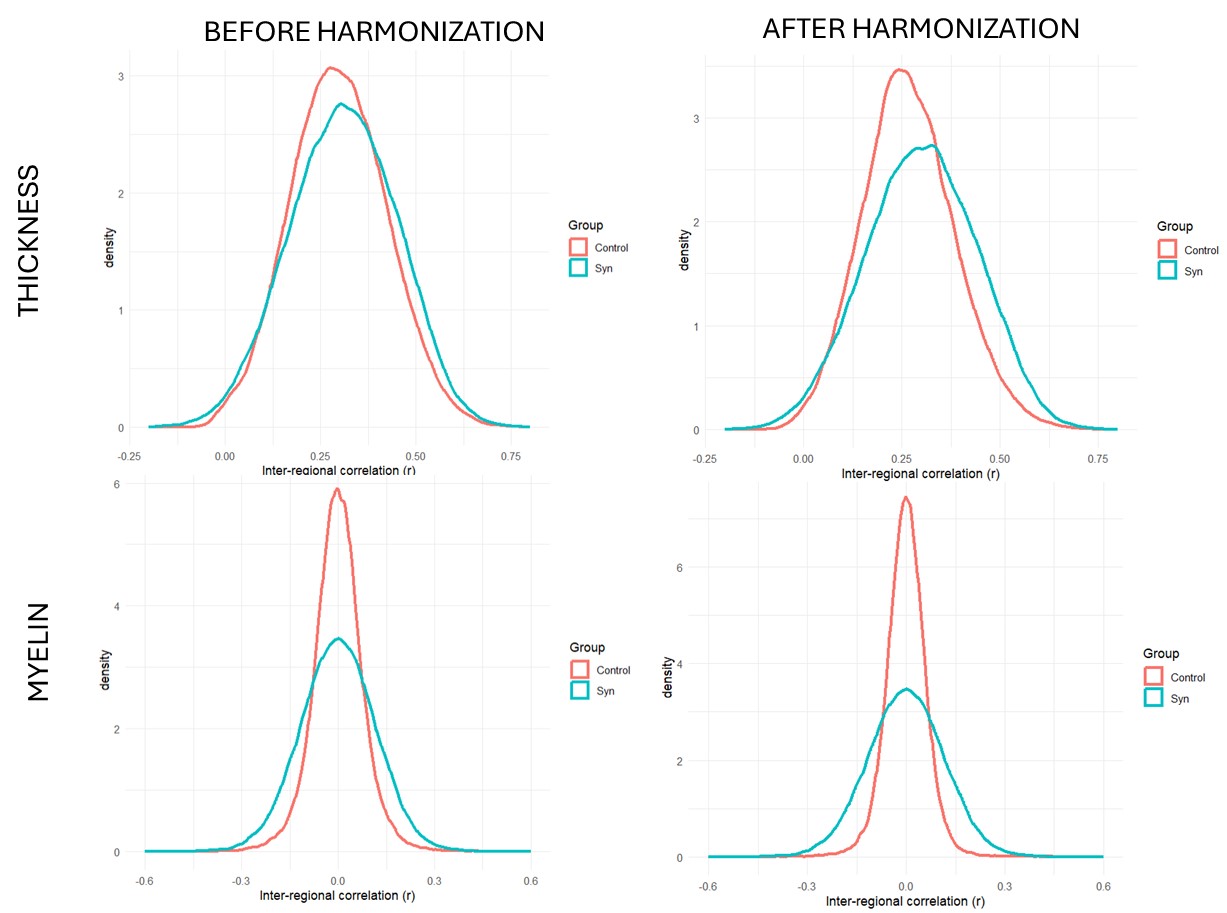
**

*Figure S2. Inter-regional correlation distributions for synaesthetes (turquoise) and controls (orange) for thickness (top) and myelin (bottom) before and after harmonization.*

**Stability of Group Differences (Synaesthetes vs. Controls) across Control Samples**

For each biomarker we calculated Cohen’s d effect sizes contrasting synaesthetes against different control datasets, and the locally acquired data from University of Sussex Clinical Imaging Sciences Centre, CISC, on the same scanner as that used for synaesthestes). For a given biomarker (e.g. N = 360 parcels), we then ascertained whether the pattern of group differences are related (Pearson’s correlation). This was done twice: once for non-harmonized data (Table S3) and once after harmonization (Table S4).

Without harmonization, there is already good evidence (r-values around 0.4) that the pattern of effect sizes is generally stable across control samples. But harmonization has a substantial effect on this and, effectively, removes almost all differences across control samples (with r-values approaching 1).

*Table S3. Before harmonization. Correlations between effect size estimates (per region or paired region) depending on which control group is compared against synaesthetes. Note that appropriate resting state data (ICA-FIX) was unavailable for HCP YA downloaded data. Critical r for N = 360 (p < .05) is r > 0.104*

| **Biomarker** | **Sussex controls v.**  **HCP YA controls** | **Sussex controls v.**  **HCP DA controls** | **HCP YA controls v.**  **HCP DA controls** |
| --- | --- | --- | --- |
| surface area (%) | 0.242 | 0.353 | 0.48 |
| subcortical volumes (%) | 0.236 | 0.579 | 0.42 |
| Thickness | 0.5 | 0.053 | 0.38 |
| thickness pairwise similarity | 0.456 | 0.162 | 0.488 |
| thickness centrality | 0.503 | 0.223 | 0.575 |
| Myelin | 0.451 | 0.384 | 0.527 |
| myelin pairwise similarity | 0.427 | 0.4 | 0.52 |
| myelin centrality | 0.415 | 0.326 | 0.46 |
| functional connectivity | NA | 0.477 | NA |
| degree centrality | NA | 0.474 | NA |
| gradient 1 | NA | 0.422 | NA |
| gradient 2 | NA | 0.485 | NA |
|  |  |  |  |
| **mean r** | **0.404** | **0.362** | **0.481** |

*Table S4. After harmonization. Correlations between effect size estimates (per region or paired region) depending on which control group is compared against synaesthetes. Note that appropriate resting state data (ICA-FIX) was unavailable for HCP YA downloaded data. Critical r for N = 360 (p < .05) is r > 0.104*

| **Biomarker** | **Sussex controls v.**  **HCP YA controls** | **Sussex controls v.**  **HCP DA controls** | **HCP YA controls v.**  **HCP DA controls** |
| --- | --- | --- | --- |
| surface area (%) | 0.967 | 0.984 | 0.979 |
| subcortical volumes (%) | 0.866 | 0.981 | 0.850 |
| Thickness | 0.930 | 0.970 | 0.908 |
| thickness pairwise similarity | 0.841 | 0.861 | 0.904 |
| thickness centrality | 0.866 | 0.876 | 0.910 |
| Myelin | 0.966 | 0.988 | 0.957 |
| myelin pairwise similarity | 0.917 | 0.933 | 0.948 |
| myelin centrality | 0.951 | 0.957 | 0.952 |
| functional connectivity | NA | 0.984 | NA |
| degree centrality | NA | 0.979 | NA |
| gradient 1 | NA | 0.870 | NA |
| gradient 2 | NA | 0.863 | NA |
|  |  |  |  |
| **mean r** | **0.913** | **0.937** | **0.926** |

**Harmonization without Group Labels**

Harmonization was performed using the dataset of Racey et al. [3] as a reference. This dataset contains known synaesthetes and known non-synaesthetes. By using this as a reference, the numerical values are unaffected by harmonization. But it is important to note that the ComBat harmonization procedure works equivalently without a reference sample and would, instead, change the numeric values of all samples. By anchoring to this reference, we also aim to future-proof the analysis pipeline to make it suitable for comparing against novel datasets.

In this exploratory analysis, the composition of the reference dataset was changed to include different proportions of synaesthetes and non-synaesthetes such that it always contained N=25 non-synaesthetes and the number of synaesthetes was either 0, 5, 10, 15, 20 or 25 (drawn at random from the larger sample). Thus, the reference sample consisted of a mixture of non-synaesthetes (majority) and synaesthetes (minority) but individuals within that group were not labelled as synaesthetes or controls. The original procedure used N=102 synaesthetes and N=25 controls in the reference sample with labelling of participants (and all other data labelled as control).

After harmonization, the biomarkers were run in exactly the same way (using the entire sample) and performance (AUC) measured. This was run on the two highest performing biomarkers (myelin and resting state functional connectivity) and one of the ‘good’ biomarkers (thickness) for comparison. The results are shown in Figure S3. The findings demonstrate that the success of the biomarkers does not hinge critically on the use of group labels during harmonization across sites, at least not at realistic estimates of synaesthesia prevalence (< 20%).

*Figure S3. Classifier performance (based on the full sample of synaesthetes and controls) but varying the composition of the reference sample during harmonization. The original method (in the main paper) involved using all data from Racey et al. as a reference sample with individuals labelled as synaesthete or control. The alternative method used a mixed sample of N=25 non-synaesthetes plus N = 0 to 25 synaesthetes as a reference (with no labelling applied to the harmonization).*

**Appendix 3: Detailed Reporting of Biomarker Performance**

**Biomarker Development Stages**

Our pre-registration included biomarker development in multiple stages following Woo et al. [4]:

- *Discovery*. This involved tuning a hyper-parameter (the number of variables to randomly sample as candidates at each split in the decision tree) with N=25 synaesthetes and a subset of controls.
- *Demonstration*. This involved taking the optimal model from the discovery phase and applying it to a new set of N=25 synaesthetes (matched closely to the discovery sample) and the remainder of the controls. Good performance at this stage implies that there was little or no over-fitting at the discover stage. The combined sample (N=50 synaesthetes, all controls) is then used to create a modified random forest biomarker (taking the same hyper-parameter as in the discovery phase).
- *Generalization*. We apply the biomarkers from the demonstration phase to a new set of synaesthetes (N=52) with somewhat different characteristics, namely a larger spread in the distribution in the number of types of synaesthesia (including only 1 or 2 types).
- *Whole sample*. This is the data reported in the main paper and reproduced here for comparison. It is based on biomarker performance from all synaesthetes (N=102) and all controls with 10-fold cross-validation and the same hyper-parameter as the discovery phase. (Note this is an extension to our pre-registration and was include because it offers the simplest summary of the data, and it broadly captures the performance of the biomarker at the other stages.)

*Table S3. Performance of the biomarkers at different stages of development (Discovery, Demonstration, Generalization, Whole Sample) in terms of specificity (probability of classifying a control as a control), sensitivity (probability of classifying a synaesthete as a synaesthete), and AUC (area-under-curve).*

|  | **Discovery** | | | **Demonstration** | | **General-isation** | **Whole sample** |
| --- | --- | --- | --- | --- | --- | --- | --- |
| **Biomarker** | **Spec.** | **Sens.** | **AUC**  **(SD)** | **Spec.** | **Sens.** | **Sens.** | **AUC (SD)** |
| surface area (%) | 0.683 | 0.840 | 0.812 (0.079) | 0.660 | 0.600 | 0.654 | 0.852 (0.052) |
| eTIV and global SA | 0.611 | 0.720 | 0.677 (0.139) | 0.596 | 0.503 | 0.640 | 0.68 (0.062) |
| subcortical volumes percent | 0.580 | 0.720 | 0.695 (0.095) | 0.517 | 0.880 | 0.558 | 0.715 (0.051) |
| thickness | 0.609 | 0.560 | 0.692 (0.037) | 0.593 | 0.760 | 0.615 | 0.809 (0.061) |
| thickness pairwise similarity | 0.691 | 0.760 | 0.763 (0.101) | 0.700 | 0.560 | 0.712 | 0.849 (0.052) |
| thickness centrality | 0.697 | 0.600 | 0.724 (0.064) | 0.610 | 0.480 | 0.577 | 0.777 (0.037) |
| myelin | 0.794 | 0.840 | 0.927 (0.038) | 0.797 | 0.880 | 0.885 | 0.968 (0.006) |
| myelin pairwise similarity | 0.774 | 0.720 | 0.861 (0.055) | 0.803 | 0.800 | 0.769 | 0.898 (0.022) |
| myelin centrality | 0.663 | 0.760 | 0.816 (0.084) | 0.627 | 0.720 | 0.769 | 0.854 (0.04) |
| functional connectivity | 0.985 | 1.000 | 1 (0) | 1.000 | 1.000 | 1.000 | 1 (0) |
| degree centrality | 0.635 | 0.760 | 0.771 (0.125) | 0.697 | 0.680 | 0.865 | 0.87 (0.046) |
| gradient 1 | 0.670 | 0.800 | 0.817 (0.116) | 0.570 | 0.680 | 0.596 | 0.753 (0.04) |
| gradient 2 | 0.660 | 0.640 | 0.749 (0.1) | 0.627 | 0.720 | 0.577 | 0.78 (0.039) |

**Variable Importance as Determined by the VSURF Package**

The R package, VSURF (Variable Selection using Random Forests) ranks the variables by their degree of importance for classification [5] and we report, for interpretation purposes, those features that are not eliminated in the first step (which removes important but redundant features).

*Table S4. Variables selected at the ‘Interpretation’ stage of the VSURF algorithm. Note that most variable names correspond to regions with the HCP parcellation atlas [6].*

| **Biomarker** | **Variables selected** |
| --- | --- |
| surface area (%) | L_IP1, R_V1, R_PFt, L_LO1, L_OP2-3, L_9-46d, L_RI, L_V3A, L_PGs |
| eTIV and global SA | eTIV, global_SA, Sex |
| subcortical volumes (%) | Right_Hippocampus, Right_Cerebellum, Cortex, Left_Cerebellum_Cortex, Left_Amygdala, Brain_Stem, Right_VentralDC, Left_Hippocampus, Right_Amygdala, Left_VentralDC, Right_Inf_Lat_Vent, CC_Central, Right_Caudate, Left_Accumbens_area, Left_Cerebellum_White_Matter, Right_Pallidum, Left_Thalamus_Proper, Right_Cerebellum_White_Matter, CSF |
| Thickness | L_V7, R_6mp, L_PFm, R_8BM, R_V3 |
| thickness pairwise similarity | Not applicable (due to use of PCA) |
| thickness centrality | L_V7 R_8BM R_V3 R_6mp L_3b R_TE1a R_IP0 R_AIP L_6d R_TE1p L_47l L_V3CD |
| Myelin | R_pOFC L_pOFC R_Pir R_a47r L_47s R_PGi R_Ig R_STGa R_p10p |
| myelin pairwise similarity | Not applicable (due to use of PCA) |
| myelin centrality | R_a47r L_47s R_STGa R_TA2 R_pOFC R_Ig R_p10p L_31pv R_13l |
| functional connectivity | Not applicable (due to use of PCA) |
| degree centrality | L_PGs R_8BM L_5L L_LO2 R_V7 L_TF L_8BM R_PIT R_9m L_24dv L_V4 L_47l |
| gradient 1 | R_STSdp L_STSdp L_STGa L_AVI R_p32 R_8Ad L_9a L_SFL L_10r L_55b |
| gradient 2 | R_p32 R_STSdp L_AVI L_STSdp L_9a R_8BM R_8Ad R_5mv L_10r L_STGa L_FOP5 R_AVI R_TE2p L_SFL R_Ig L_BA43 L_EC R_TPOJ1 R_OP4 |
| Behavioural variables (N=35) | Creative novelty, Attention-to-detail (AQ), Openness to Experience (personality), Sensory sensitivity (GSQ), Overall creativity, Intelligence (Ravens), and Attention Switching (AQ) |

**Appendix 4: Further Details of Behavioural Results**

Table S5 reports the full descriptive and inferential results, as per the pre-registration, which are summarised in the main document. The observed data for calculating Bayes Factors is the difference in means between the groups and the standard error (SE) of the difference. The prior was modelled using the room-to-move heuristic [7] and a half-normal distribution (assuming that the direction of the difference favours synaesthetes). This heuristic makes the assumption that group differences that are small in size are more probable than larger differences and, moreover, that the ceiling of any such difference is the ceiling of the scale itself. Specifically, the distance between the control mean and the scale ceiling is operationalised as two standard deviations of the half-normal. Table S6 reports exploratory (not pre-registered) analyses of the clinical data in which continuous data is binarized using previously published norms.

Figure S4 shows a visualization of the IES-R (Impact of Events Scale – Revised) which was motivated by the finding that Projectors versus Associators differ on this measure (noting that this is the only measure where a difference was found). Figure S5 shows a visualization of the number of types of synaesthesia against all dependent variables, and this should be regarded as post-hoc and exploratory (our pre-registration specified a contrast between many (4+) versus few (1-3) types, as reported in the main paper).

Figure S6 explores differences within synaesthetes by splitting the sample according to the presence/absence of different types of synaesthesia. This analysis was pre-registered. The results point to possible behavioural differences amongst types of synaesthesia that are beyond the scope of exploration here (for example, splitting the synaesthetes in terms of the presence-absence of sequence-space reveals no significant differences between sub-groups whereas other splits are significant). Further analyses, would need to disentangle this from the number of types of synaesthesia.

*Table S5: Descriptive and inferential statistics for the N = 35 clinical and cognitive dependent variables. The code for generating the data in the table was written in R and is available in the main OSF repository.*

|  | **Variable** | **Syn mean (SD)** | **Control mean (SD)** | **Cohen’s d** | **t-values** | **p values** | **p values**  **FDR** | **Bayes**  **Factor** |
| --- | --- | --- | --- | --- | --- | --- | --- | --- |
| Hyper-mobility | Mob. | 1.734  (1.493) | 1.294  (1.349) | 0.308 | 2.387 | 0.018 | 0.057 | 1.697 |
| Autism Quotient, AQ | At.Sw | 5.789  (2.760) | 4.716  (2.400) | 0.411 | 3.202 | 0.002 | 0.008 | 21.304 |
|  | At.De | 6.695  (2.247) | 4.771  (2.312) | 0.843 | 6.471 | 0.000 | 0.000 | 1.337x10^8^ |
|  | Com. | 3.180  (2.582) | 2.716  (2.182) | 0.192 | 1.500 | 0.135 | 0.263 | 0.252 |
|  | Ima. | 2.773  (2.040) | 2.486  (1.757) | 0.150 | 1.165 | 0.245 | 0.452 | 0.089 |
|  | So.Sk | 3.422  (2.725) | 3.440  (2.713) | -0.007 | -0.052 | 0.958 | 0.958 | 0.070 |
| Sensory sensitivity | GSQ | 60.305  (24.958) | 46.239  (20.802) | 0.606 | 4.732 | 0.000 | 0.000 | 3577 |
| Anxiety ASI-3 | Cog. | 5.945  (5.748) | 5.404  (5.721) | 0.094 | 0.725 | 0.469 | 0.684 | 0.058 |
|  | Phys. | 6.313  (5.465) | 6.239  (5.723) | 0.013 | 0.101 | 0.919 | 0.946 | 0.025 |
|  | Soc. | 10.094  (5.998) | 9.431  (6.112) | 0.109 | 0.839 | 0.402 | 0.653 | 0.137 |
| DASS-21 | Anx. | 4.211  (4.266) | 3.385  (3.237) | 0.215 | 1.691 | 0.092 | 0.190 | 0.227 |
|  | Str. | 7.586  (4.945) | 6.541  (4.124) | 0.227 | 1.773 | 0.077 | 0.169 | 0.356 |
|  | Dep. | 5.453  (4.944) | 5.706  (4.852) | -0.052 | -0.397 | 0.692 | 0.813 | 0.013 |
| IES-R (PTSD) | Av. | 10.898  (8.301) | 10.110  (8.028) | 0.096 | 0.742 | 0.459 | 0.684 | 0.077 |
|  | H.Ar | 5.461  (6.453) | 5.239  (6.000) | 0.035 | 0.275 | 0.784 | 0.857 | 0.072 |
|  | Int | 10.563  (8.769) | 10.028  (8.318) | 0.062 | 0.481 | 0.631 | 0.788 | 0.057 |
| Personality, BFI-2 | Extra. | 38.391  (9.776) | 37.716  (9.213) | 0.071 | 0.547 | 0.585 | 0.788 | 0.071 |
|  | Agr. | 46.898  (6.585) | 46.450  (7.019) | 0.066 | 0.505 | 0.614 | 0.788 | 0.119 |
|  | Con. | 42.109  (9.306) | 41.633  (9.443) | 0.051 | 0.390 | 0.697 | 0.813 | 0.074 |
|  | Neg.E | 38.281  (10.879) | 37.064  (11.692) | 0.108 | 0.825 | 0.411 | 0.653 | 0.124 |
|  | Op.Ex | 50.617  (7.197) | 45.220  (7.905) | 0.714 | 5.458 | 0.000 | 0.000 | 359534 |
| AUT Creativity | Nov. | 2.025  (0.121) | 1.962  (0.093) | 0.571 | 4.486 | 0.000 | 0.000 | 587 |
|  | Cre. | 2.932  (0.273) | 2.831  (0.271) | 0.370 | 2.846 | 0.005 | 0.019 | 7.999 |
| Memory | Ro | 0.153  (0.188) | 0.149  (0.172) | 0.023 | 0.177 | 0.860 | 0.912 | 0.012 |
|  | F | 1.131  (0.486) | 1.077  (0.460) | 0.113 | 0.854 | 0.394 | 0.653 | 1.177 |
|  | AUC | 0.792  (0.094) | 0.785  (0.090) | 0.081 | 0.624 | 0.533 | 0.746 | 0.039 |
|  | Ac.Con | 0.230  (0.183) | 0.238  (0.156) | -0.043 | -0.334 | 0.739 | 0.834 | 0.039 |
| PSI-Q Mental Imagery | Bod.Sen | 37.945  (9.752) | 34.266  (9.927) | 0.373 | 2.867 | 0.005 | 0.019 | 9.442 |
|  | Emo. | 36.164  (9.975) | 33.569  (9.307) | 0.267 | 2.070 | 0.040 | 0.104 | 1.256 |
|  | Snd. | 39.688  (8.509) | 37.073  (8.700) | 0.303 | 2.329 | 0.021 | 0.061 | 2.492 |
|  | Tst. | 35.844  (11.054) | 33.321  (10.398) | 0.234 | 1.808 | 0.072 | 0.168 | 0.815 |
|  | Tch. | 39.953  (8.444) | 36.138  (9.634) | 0.422 | 3.215 | 0.002 | 0.008 | 28.276 |
|  | Vis. | 40.852  (7.808) | 38.083  (8.601) | 0.337 | 2.576 | 0.011 | 0.037 | 4.881 |
|  | Sm. | 34.531  (11.469) | 31.550  (10.913) | 0.265 | 2.047 | 0.042 | 0.104 | 1.228 |
| Matrices | Mat. | 7.406  (2.366) | 5.908  (2.463) | 0.619 | 4.752 | 0.000 | 0.000 | 9666 |

*Table S6. Reanalysis of clinical measures after applying a binarizing, clinically validated, cut-off. Note that cut-offs for the DASS-21 offs are based on multiplying the observed scores x 2 as is conventionally done to compare the 21-item against 42-item norms [8]. On many items (e.g. ASI-2, DASS) we find unusually high scores in both groups, relative to published norms, which may relate to the timing of data collection. We note that data collection was carried out during the COVID-19 pandemic (between November 2020 and March 2022) and other studies using the same measures during this time also report similar mean scores [9].*

| **Measure including cut-off source** | **Clinical cut-off** | **Cases (Syns)** | **Cases**  **(Non-syns)** | **Significance** | **Effect size**  **(odds ratio)** |
| --- | --- | --- | --- | --- | --- |
| Autism quotient [10] | ≥ 32 | 23/128 (18.0%) | 6/109 (5.5%) | Χ^2^(1) = 8.516,  p = .004* | 3.760 |
| DASS Stress [8] | ≥ 26 (severe) | 22/128 (17.2%) | 9/109 (8.3%) | Χ^2^(1) = 4.130,  p = .042* | 2.306 |
| Hypermobility [11] | ≥ 2 | 66/128 (51.6%) | 39/109 (35.8%) | Χ^2^(1) = 5.943,  p = .015* | 1.911 |
| DASS Anxiety [8] | ≥ 15 (severe) | 25/128 (19.5%) | 15/109 (13.8%) | Χ^2^(1) = 1.397,  p = .237 | 1.521 |
| Anxiety Sensitivity Index [12] | ≥ 23 (high) | 58/128 (45.3%) | 46/109 (42.2%) | Χ^2^(1) = 0.231,  p = .631 | 1.135 |
| Impact of Event Scale [13] | ≥ 34 | 42/128 (32.8%) | 33/109 (30.3%) | Χ^2^(1) = 0.175,  p = .676 | 1.125 |
| DASS Depression [8] | ≥ 21 (severe) | 19/128 (14.8%) | 21/109 (19.3%) | Χ^2^(1) = 0.821,  p = .365 | 0.730 |

*Figure S4: Mean subscale scores (+/- 1 SEM) for Projectors and Associators (both with grapheme-colour synaesthesia) for the IES-R which is an informal measure of PTSD-like symptoms in response to a recalled stressful event. For comparison, the means for our non-synaesthete sample were 10.1 (Avoidance), 5.2 (Hyperarousal), and 10.0 (Intrusion). In terms of meeting a clinical cut-off (total score ≥ 34) this was achieved for 56% of Projectors (9/16), 28% of Associators (20/72) and 32% of non-synaesthetes (35/109).*


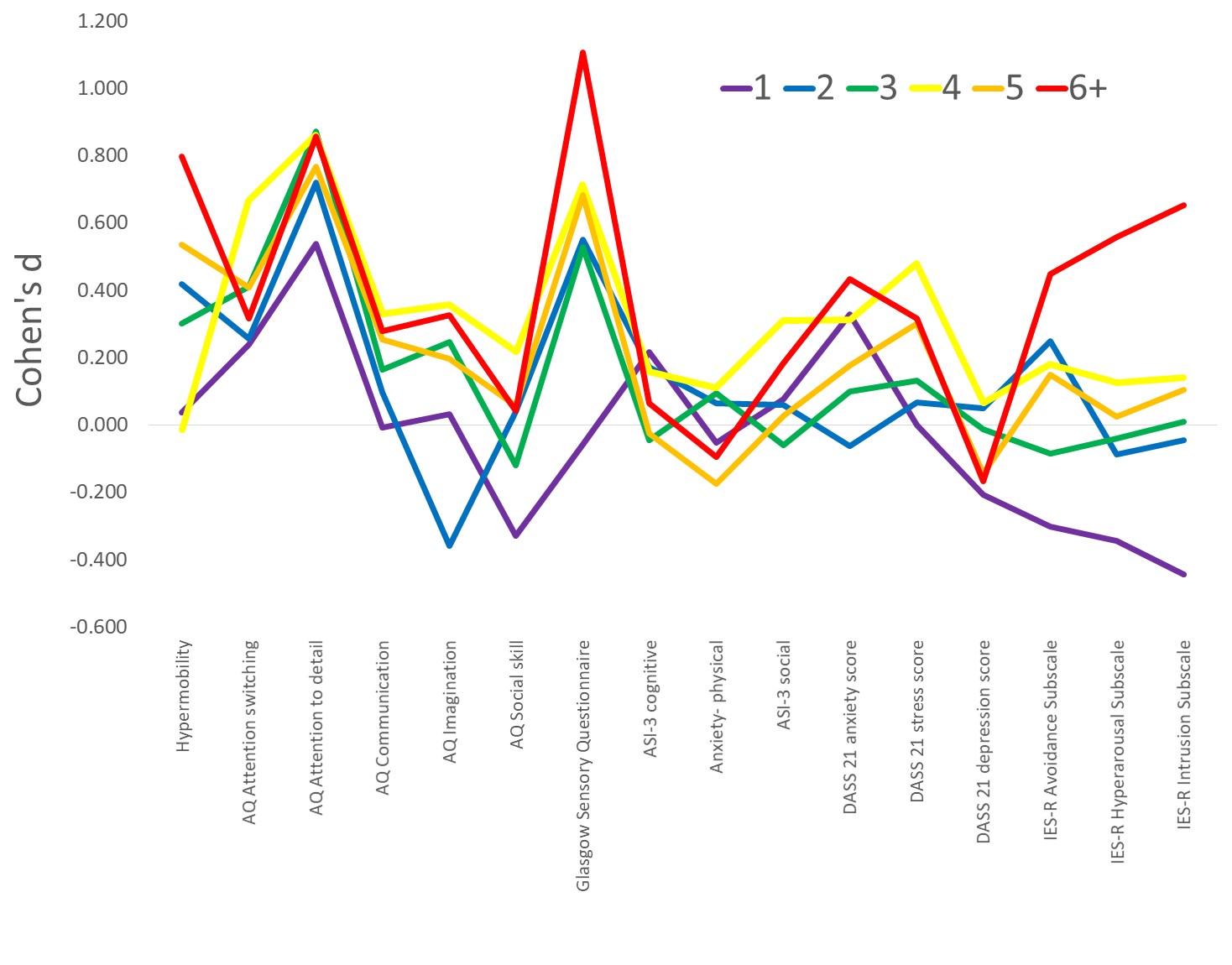


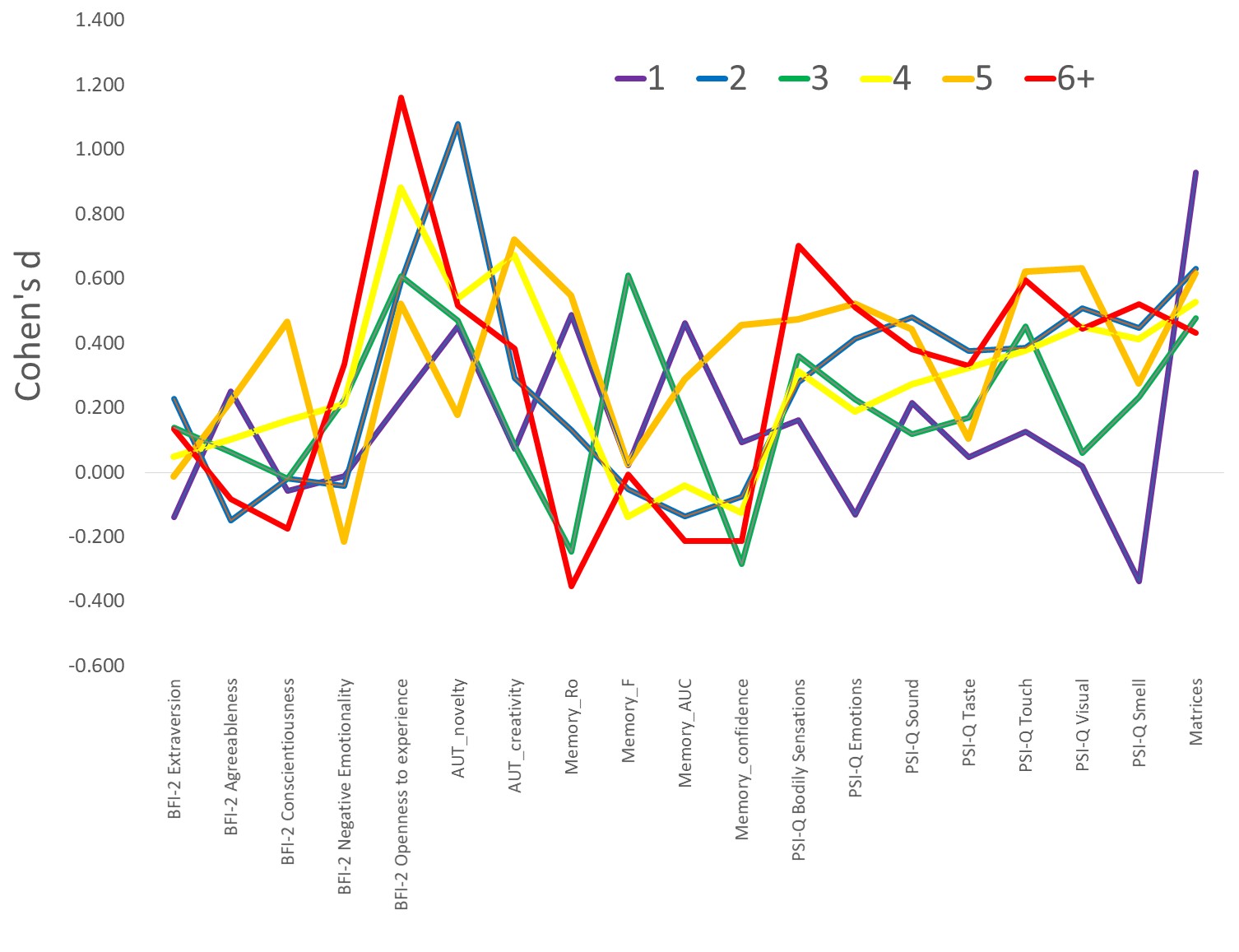


*Figure S5. Effect sizes (Cohen’s d) contrasting synaesthetes (with 1 to 6+ types) against non-synaesthetes. The top graph shows the clinically related dependent variables, and the bottom graph shows those variables related to personality and cognition. Note: If there is a relationship between number of types and magnitude of effect then the data would be aligned as a ‘spectrum’ (red at top, purple at bottom).*


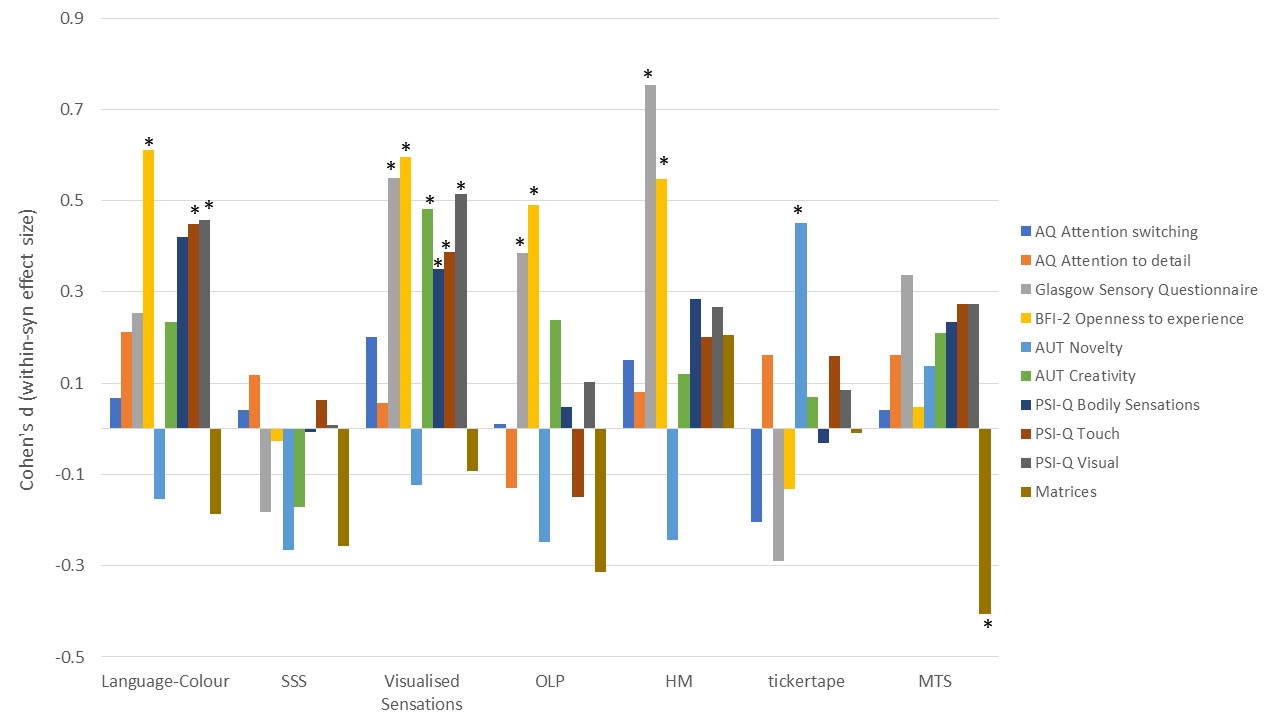


*Figure S6: Behavioural differences splitting the synaesthesia group by type. For example, splitting by the presence (N=100) or absence (N=28) of Language-colour and calculating the effect size (Cohen’s d) for that split. Here we comply with our preregistered analysis of including only synaesthesia types with 20 or more cases, and only including variables with a sensitive (BFs > 3) group difference between synaesthetes and controls. As this analysis is exploratory we did not correct for multiple comparisons. * p < .05, uncorrected*

**Appendix 5: Inter-regional Correlations - Replication of Hänggi et al. (2011) and Extension to Intracortical Myelin**

If one takes pairs of cortical regions, one can examine whether individual differences in the properties of one region are correlated with individual differences in another region (e.g., is variation in the thickness of region A correlated with variation in the thickness of region B?). These correlations have been taken as indicative markers of coordinated neurodevelopment between pairs of regions [14], using dependent measures such as cortical thickness [15] and cortical myelination [16]. These patterns of covariation track other structural differences (e.g., tractography) and functional differences (e.g., resting state networks).

Hänggi et al. [17] reported that synaesthetes have stronger correlations of cortical thickness between pairs of regions than controls. The biomarker reported in the present paper was developed along these lines but using absolute pairwise differences in thickness (mm) rather than r-values. This is necessary because r-values are a between-subject variable, but we need a within-subject variable for a biomarker approach [18]. In this supplementary section, we attempt a more direct replication of the main analyses of Hänggi et al. [17] using their approach. We also extend it from thickness to our measure of intracortical myelin.

**THICKNESS**

Hanggi et al. (2011) state: “parcellations were used to construct the association (connectivity) matrix (Aij) based on the cortical thickness correlation matrix (Cij) between all pairs of parcellations, resulting in 154 × 154 association matrix (network) for the synesthetes and one for the nonsynesthetes.”

We apply the same method using the HCP derived parcellations which generates a 360 x 360 association matrix (r values) for each group. A high value indicates that individual differences in the cortical thickness of this pair of brain regions are correlated.

Figure S7 shows the correlation matrices for synaesthetes (top) and controls (bottom). Note that almost all values are positive going (depicted in blue) or close to zero (depicted in white), across both groups. The main diagonal (showing r = 1) is not informative, but the other visible diagonals represent correlations between the same regions across hemispheres.


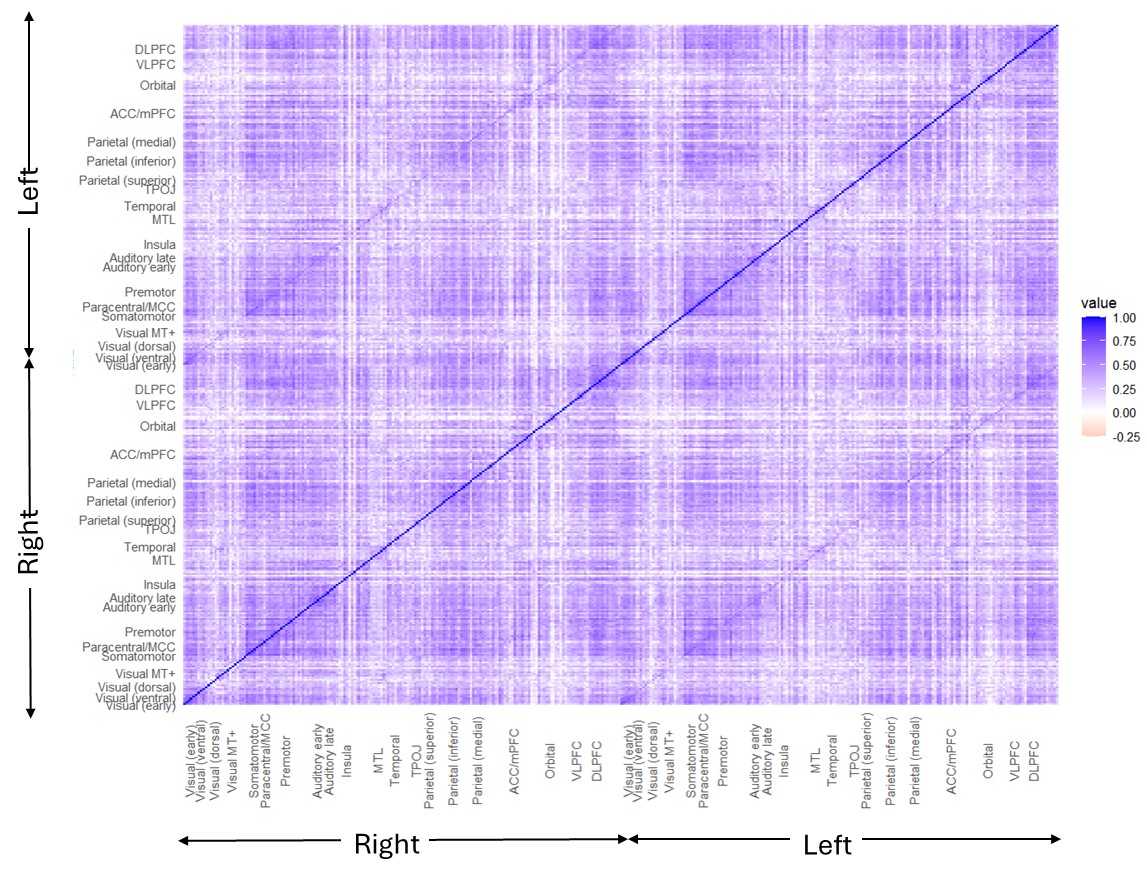


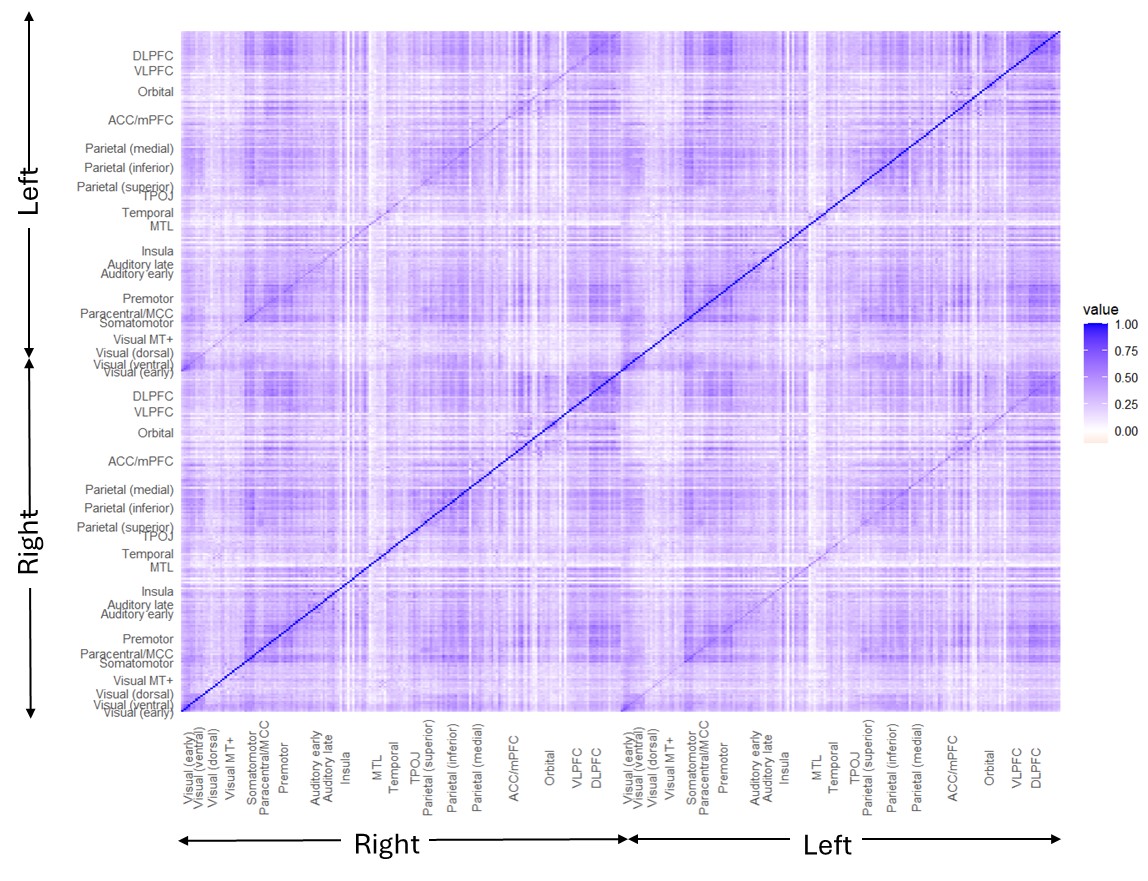


*Figure S7. Correlations amongst pairs of regions (360 x 360) in their cortical thickness profile for synaesthetes (top) and controls (bottom).*

Figure S8 shows the difference map in the r values (synaesthetes – controls). There are differences in both directions: greater inter-regional thickness correlations in synaesthetes relative to controls (shown in blue) and greater inter-regional thickness correlations in controls relative to synaesthetes (shown in red). The latter shows four prominent red clusters corresponding primarily to a set of frontal lobe regions (regions 55-91 in the list of regions in the HCP atlas, from roughly area 6d to area 10pp) which show stronger inter-regional correlations in controls than synaesthetes (both within and between hemispheres).


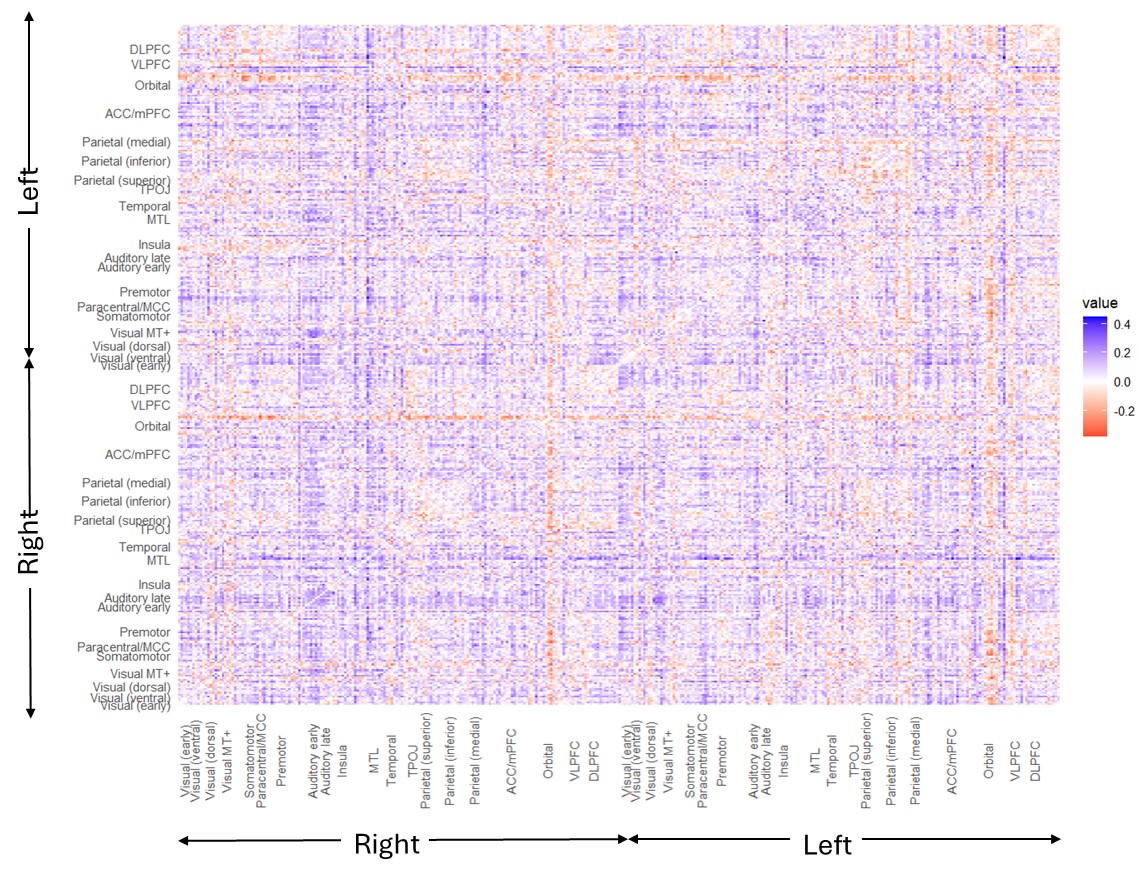


*Figure S8. Differences in the r values, contrasting synaesthetes minus controls. Note that there are differences in both directions but more positive going differences overall (in blue).*

Hanggi et al. (2011) state: "Networks (graphs) Gr were represented by weighted matrices (Aij, one matrix for each correlation threshold r) with N nodes and Kr edges, where nodes represent cortical regions and edges represent the weighted connections between these cortical regions. There is currently no definitive and generally accepted strategy for applying a particular threshold… Each connectivity matrix was therefore thresholded repeatedly over a wide range of correlation thresholds in increments of r = 0.025 from r = 0.15 to r = 0.525, resulting in 16 networks with different connection densities per group."

This approach was applied to this dataset applying the r thresholds from their paper to our data. In brief, this involves counting all correlations above the threshold, and dividing by the total number of correlations in the dataset (= 360x359/2, here just taking the above-diagonal values as the matrix is symmetric). This gives a value of connection density. The distribution is shown in Figure S9. The higher connection density for synaesthetes across a range of thresholds replicates Hanggi et al. (2011).

*Figure S9. Connection density (i.e. proportion of connections above threshold) as a function of correlation strength. Here, the connections are summed across all regions. A comparable measure (degree centrality) divides these across the 360 regions. The two sets of 360 regions from each group can be compared via t-tests (see below).*

Hanggi et al. (2011) state: “Degree centrality is the sum of weights incident upon a node (i.e., the sum of weights of the edges that a node has). Degree is often interpreted in terms of the capability of a node to catch whatever is flowing through the network… In each differently thresholded 2366-node network, mean (across nodes) degree centrality scores were significantly increased in synesthetes compared with nonsynesthetes (all p ≪ 0.0001, Bonferroni corrected p = 0.05/16 = 0.003).”

Degree centrality measures (at each r threshold) are the sum of the number of connections above each threshold (1, 0) at each node. This gives a set of 360 centrality measures for synaesthetes, and 360 centrality measures for controls which can be compared with a paired t-test (pairing across nodes). (The average degree centrality is the same as the overall connection density, as shown in Figure S9). Applying the same Bonferonni correction (p < .003), we find significantly higher degree centrality in synaesthetes at threshold values of r = 0.175 and above.

*Table S7. Group differences in degree centrality (across 360 regions/nodes) depending on different r-value thresholds for determining whether a connection is present or not.*

| **r threshold** | **t_values** | **p_values** |
| --- | --- | --- |
| 0.15 | 1.500536 | 0.134 |
| 0.175 | 3.396692 | 0.001 |
| 0.2 | 5.727494 | 0.000 |
| 0.225 | 8.768345 | 0.000 |
| 0.25 | 11.67779 | 0.000 |
| 0.275 | 14.58132 | 0.000 |
| 0.3 | 16.89806 | 0.000 |
| 0.325 | 18.83144 | 0.000 |
| 0.35 | 19.54465 | 0.000 |
| 0.375 | 19.6246 | 0.000 |
| 0.4 | 19.01521 | 0.000 |
| 0.425 | 17.80163 | 0.000 |
| 0.45 | 16.68145 | 0.000 |
| 0.475 | 15.52723 | 0.000 |
| 0.5 | 14.32408 | 0.000 |
| 0.525 | 12.62558 | 0.000 |

Summary

The key findings of Hanggi et al. (2011) are replicated insofar as we demonstrate greater inter-regional correlations of cortical thickness in synaesthetes relative to controls. The results do not support the conclusion that these effects are ‘global’ (i.e., found in all pairwise comparisons) – we find parts of the brain showing the reverse pattern. However, the results generally support the notion of widespread differences throughout the brain as opposed to highly local differences (e.g., relating to certain sensory regions such as V4).

**MYELIN**

Applying the analysis method used above to myelin, produces very different results both within and between groups. Unlike in the comparable visualisations for cortical thickness, there are also negative correlations (indicated in red) such that individual differences linked to higher myelin in one region are linked to individual differences in lower myelin in another. In other words, individual differences in cortical myelin variation are linked to a ‘pull and push’ mechanism whereas cortical thickness variation appears to have a ‘pull’ mechanism alone (all correlations are positive, albeit differing in degree).

In terms of visualization – Figure S10 - the control data appears to have more structure than the synaesthete data. Specifically, horizontal and vertical lines (red, white and blue) in the control data are indicative of consistent inter-regional correlations. The control dataset has more correlations closer to zero (indicated by white).

The synaesthete pattern appears to have less structure as indicated (in visualization) by fewer groupings of red, white and blue into lines and squares. The correlations for synaesthetes tend to deviate more from zero: in terms of visualization the colours are darker.


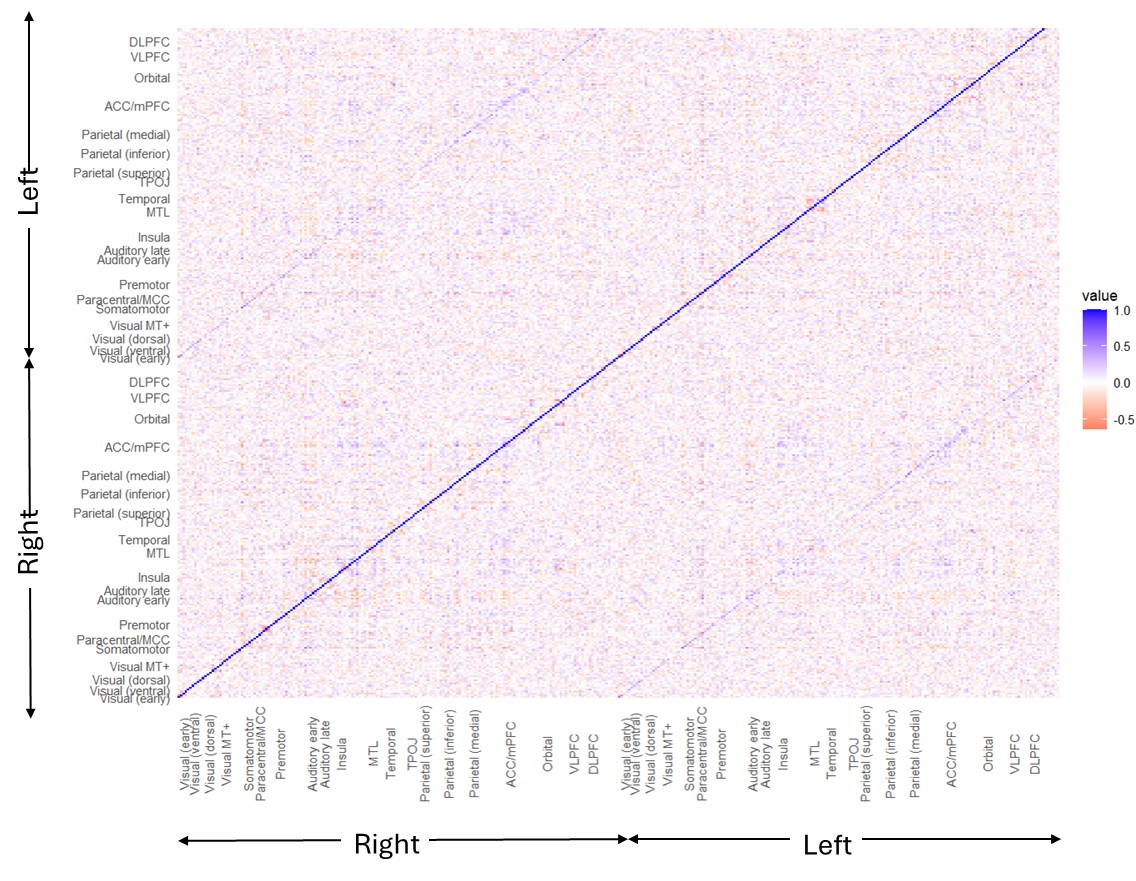


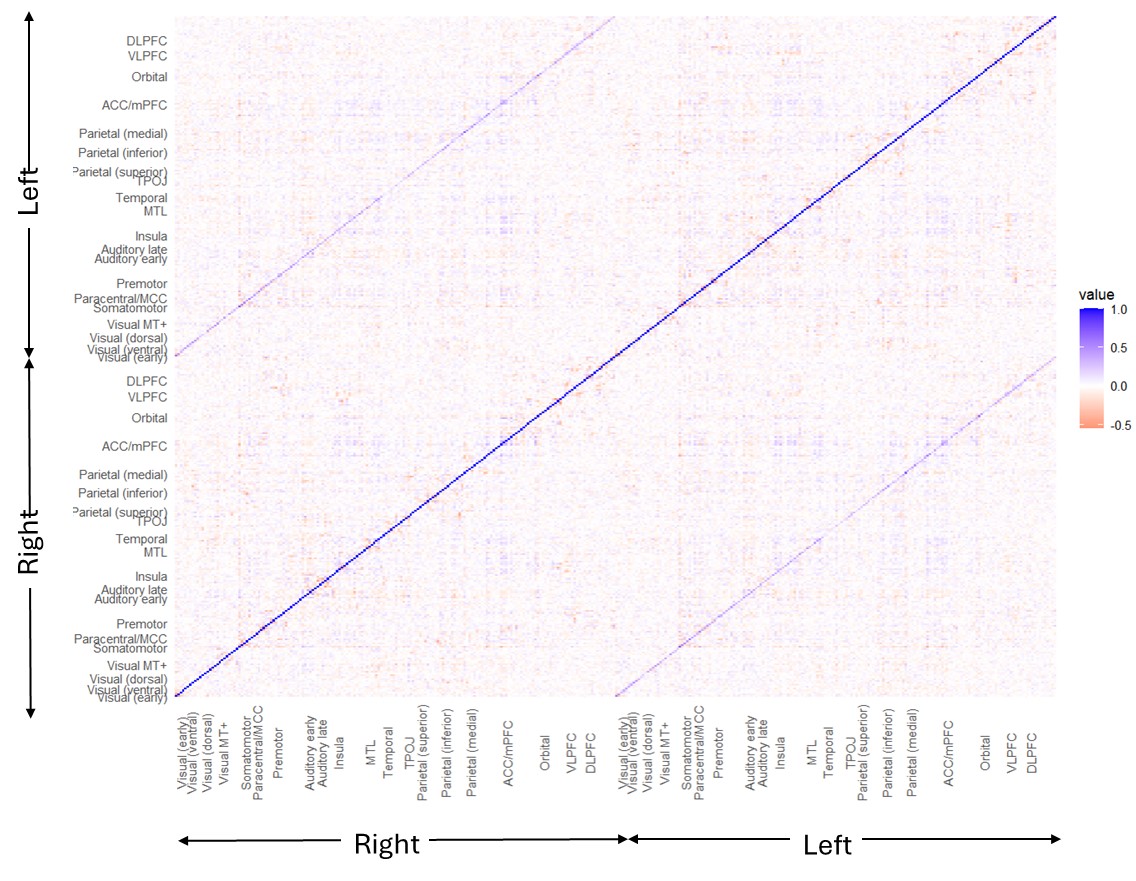


Figure S10. *Correlations amongst pairs of regions (360 x 360) in their cortical myelination profile for synaesthetes (top) and controls (bottom).*


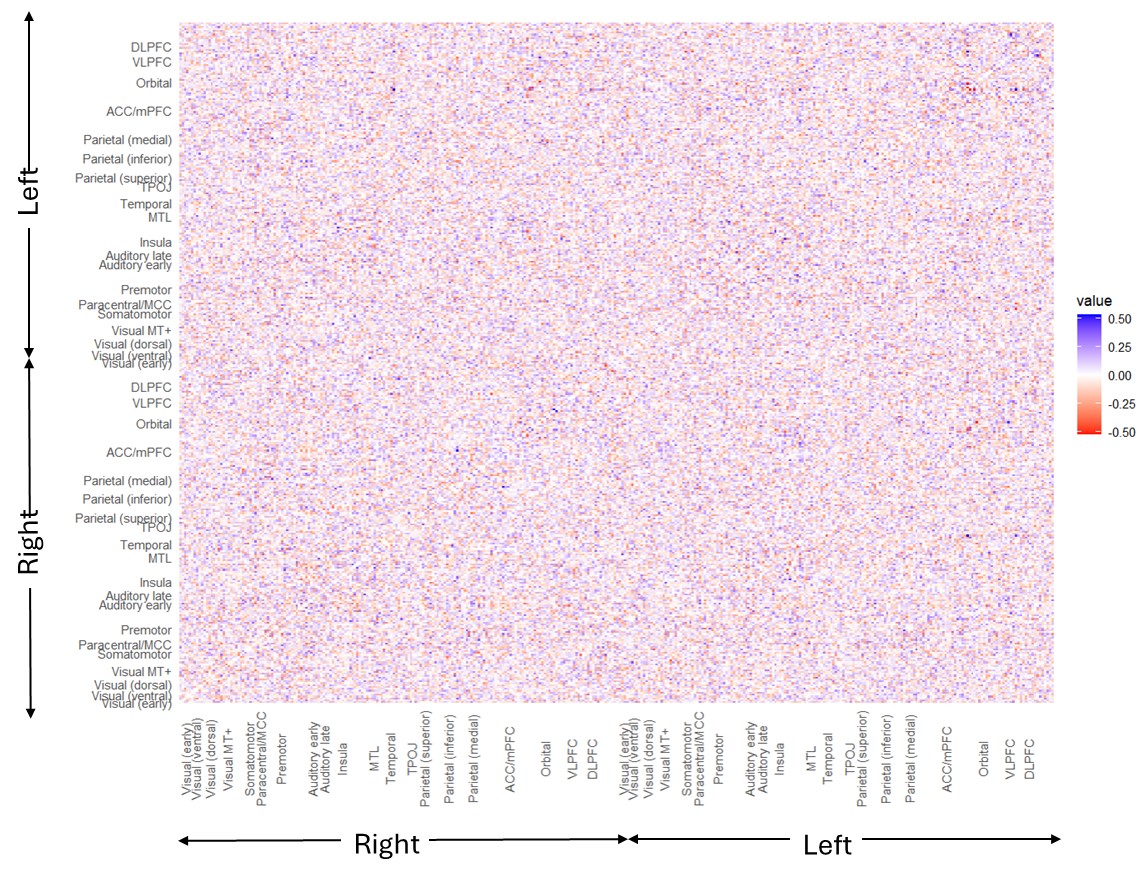


*Figure S11. The difference between these r-maps (synaesthetes minus controls). The data looks essentially unstructured according to larger scale anatomy (e.g. frontal lobes).*

Summary and Conclusions

The mechanisms that produce inter-individual correlation in cortical thickness have a ‘pull’ character, whereas those relating to cortical myelin have a ‘pull and push’ character. There is a stronger ‘pull’ mechanism driving changes in cortical thickness which results in more positive inter-regional correlations (albeit not uniformly), and a stronger ‘pull and push’ mechanism for cortical myelination (resulting in more extreme correlations in both positive and negative going directions). (Note: whilst we don’t observe any negative correlations between regions in terms of cortical thickness, we see group differences that are less positive going – i.e. relative differences in the degree to which the correlations are positive). Correlations in thickness between frontal lobe regions are notably higher in controls than synaesthetes, these regions being the latest to reach maturity.

**Appendix 6: Associations between Biomarkers**

For each biomarker, it is possible to extract a single dependent variable (per participant) that corresponds to ‘how synaesthetic’ that person is on a 0-1 scale. This corresponds to the proportion of votes in the Random Forest for classifying a person as synaesthete (where 1 means all votes are in favour of synaesthesia) or non-synaesthete (where 0 means no votes are in favour of synaesthesia). So, for example, a positive correlation between the biomarker for myelin and the biomarker for thickness would imply that people who can be classified accurately on the basis of myelin profile is associated with classification accuracy based on the thickness profile (note: it does not mean that higher thickness is linked to higher myelin).

The main analysis reported in the paper is not suited for this analysis because the use of down-sampling (due to having far more controls than synaesthetes) results in different subsamples of controls in each biomarker. The analysis was therefore repeated by taking all synaesthetes (N=102) and a random selection of N=102 controls (selecting from controls with full datasets available across all biomarkers). The same classifiers were rerun and ‘votes’ were taken from the held-out data (i.e. from the test folds rather than training set). Table S8 shows the Pearson correlations across biomarkers for the full sample of N=204 (note the critical value of r is 0.137 for p < .05).

*Table S8. Pearson’s correlations between different biomarkers according to the extent to which the same individuals are classified as being a synaesthete across the biomarkers.*

**

A set of Pearson’s correlations can be transformed into a set of partial correlations that may better represent the underlying relationship between variables. For example, a significant Pearson’s correlation between variable A and variable C might be fully explained by the intermediate variable B (in this case, the partial correlation A-C would be non-significant and those between A-B and B-C would be significant). The set of partial correlations was estimated with a Gaussian Graphical Model (GGM) in R using the qgraph package using pairwise random Markov fields [19]. Model selection was based on EBIC (Extended Bayesian Information Criteria) and the default gamma parameter of 0.5 (which controls how sparse the model is so not all nodes are fully connected). The graphical LASSO (least absolute shrinkage and selection operator) is applied for regularization, i.e., to reduce over-fitting, using the R glasso package. This shrinks all coefficients in a systematic way and sets small ones to zero [20]. The resulting set of regularized partial correlations can be visualized as a network diagram as shown in Figure S12. This gives some insight into which biomarkers are more central (i.e. have more mutual influence over other variables). The biomarker relating to pairwise inter-regional differences in myelin is notable for its connectedness to a wide range of other biomarkers.


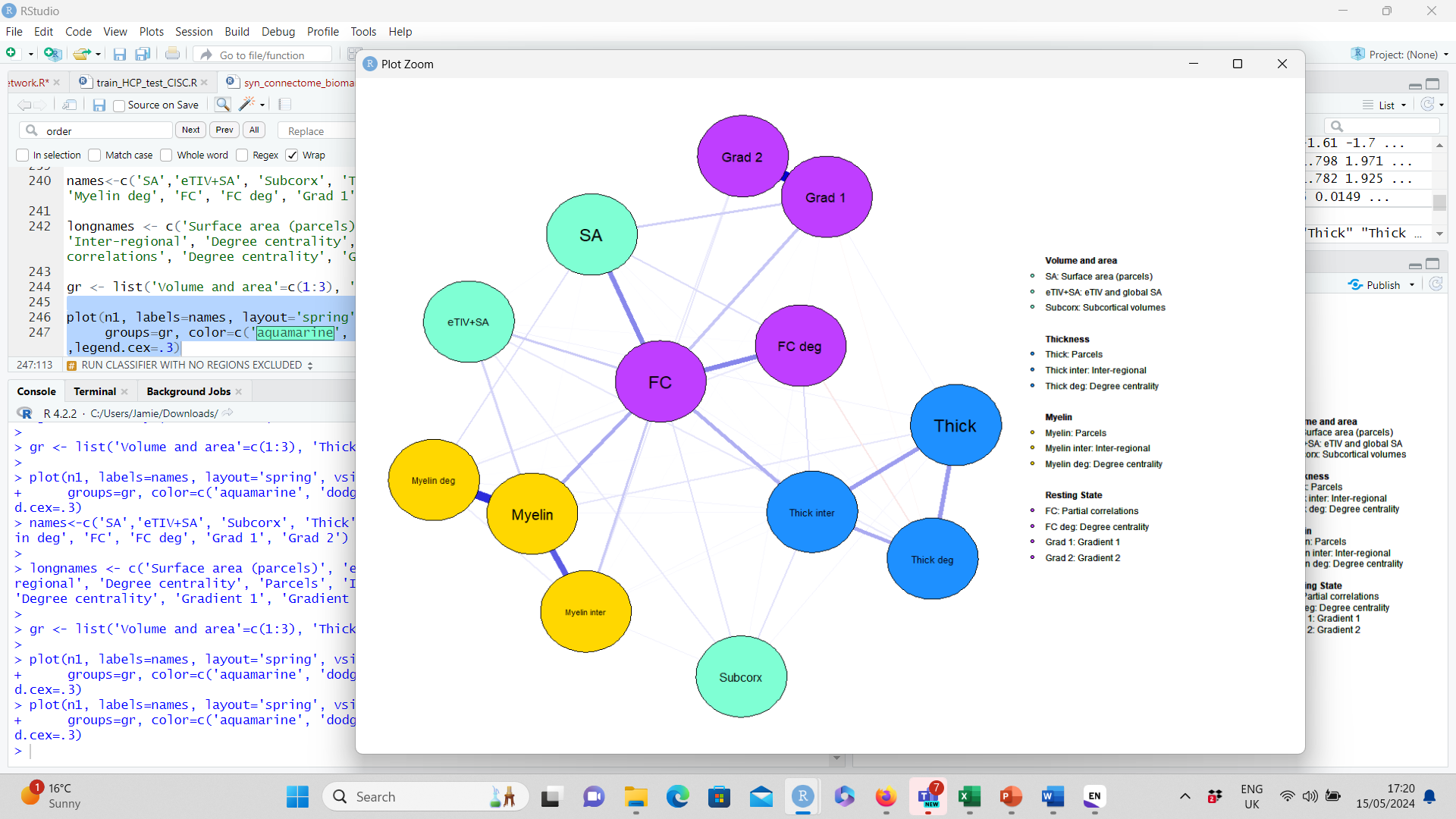


*Figure S12. A network summary of the relationship between the ability of the 13 different biomarkers to predict synaesthesia status. In interpreting the model, we advise the reader that any non-zero weighted association can be interpreted as making a significant contribution to the model. The thickness of the lines indicates the strength of the association measured as partial correlations (blue and red lines represent positive and negative associations respectively). The spatial separation, for visualization purposes, is also based on the strength of association ignoring whether the partial correlation is positive or negative (termed a spring layout; [21]), and irrespective of the biomarker type (the four colours are purely for visualization).*

**Appendix 7: Links to results files showing group differences (effect size) by region**

Column A = Region, using HCP label

Column B = Cohen’s d effect size (syns – controls) for whole sample

Column C = p-value (uncorrected) for whole sample

Column D = p-value (FDR corrected) for whole sample

Column E = p-value (uncorrected) after excluding HCP YA controls

Column F = p-value (uncorrected) after excluding HCP D/A controls

Column G = Cohen’s d effect size (syns – controls) for just local controls at CISC

Column H = Cohen’s d effect size (syns – controls) for just HCP YA controls

Column I = Cohen’s d effect size (syns – controls) for just HCP D/A controls

Column J = whether FDR significant overall and when dropping either of the two large control samples and showing the CISC controls with an effect in same direction as other control samples

| **Biomarker** | **Link** |
| --- | --- |
| surface area (%) | <https://osf.io/69nje>, biomarker1A_d_values_harm.xlsx |
| surface area (mm^2^) | <https://osf.io/52atw>, biomarker1C_d_values_harm.xlsx |
| eTIV and global SA | Not applicable (not parcellated) |
| subcortical volumes (%) | <https://osf.io/7d6se>, biomarker5A_d_values_harm.xlsx |
| subcortical volumes (mm^3^) | <https://osf.io/u5v2g>, biomarker5B_d_values_harm.xlsx |
| Thickness | <https://osf.io/34wzd>, biomarker2_d_values_harm.xlsx |
| thickness pairwise similarity | <https://osf.io/8d93u>, biomarker3A_d_values_harm.xlsx |
| thickness centrality | <https://osf.io/ju3dn>, biomarker3B_d_values_harm.xlsx |
| Myelin | <https://osf.io/ft72j>, biomarker4A_d_values_harm.xlsx |
| myelin pairwise similarity | <https://osf.io/v3atb>, biomarker4B_d_values_harm.xlsx |
| myelin centrality | <https://osf.io/63uqy>, biomarker4C_d_values_harm.xlsx |
| functional connectivity | <https://osf.io/6ty98>, biomarkerF1A_d_values_harm.xlsx |
| degree centrality | <https://osf.io/tgc3u>, biomarkerF1B_d_values_harm.xlsx |
| gradient 1 | <https://osf.io/8duaf>, biomarkerF2A_d_values_harm.xlsx |
| gradient 2 | <https://osf.io/qmgsr>, biomarkerF2B_d_values_harm.xlsx |

Note that the raw data by participant and region is available at: <https://osf.io/xbnjw/>

**References**

1. Basha, S.J., et al. *A Review on Imbalanced Data Classification Techniques*. in *2022 International Conference on Advanced Computing Technologies and Applications (ICACTA)*. 2022.

2. Horng, H., et al., *Generalized ComBat harmonization methods for radiomic features with multi-modal distributions and multiple batch effects.* Sci Rep, 2022. **12**(1): p. 4493.

3. Racey, C., et al., *An Open Science MRI Database of over 100 Synaesthetic Brains and Accompanying Deep Phenotypic Information.* Scientific Data, 2023. **10**(1): p. 766.

4. Woo, C.W., et al., *Building better biomarkers: brain models in translational neuroimaging.* Nature Neuroscience, 2017. **20**(3): p. 365-377.

5. Genuer, R., J.M. Poggi, and C. Tuleau-Malot, *VSURF: An R Package for Variable Selection Using Random Forests.* R Journal, 2015. **7**(2): p. 19-33.

6. Glasser, M.F., et al., *A multi-modal parcellation of human cerebral cortex.* Nature, 2016. **536**(7615): p. 171-+.

7. Dienes, Z., *How do I know what my theory predicts?* Advances in Methods and Practices in Psychological Science, 2019. **2**(4): p. 364-377

8. Lovibond, S.H. and P.F. Lovibond, *Manual for the Depression Anxiety Stress Scales. (2nd. Ed.)*. 1995, Sydney: Psychology Foundation.

9. Manning, K., et al., *Anxiety Sensitivity Moderates the Impact of COVID-19 Perceived Stress on Anxiety and Functional Impairment.* Cognit Ther Res, 2021. **45**(4): p. 689-696.

10. Baron-Cohen, S., et al., *The Autism-Spectrum Quotient (AQ): Evidence from Asperger syndrome/high-functioning autism, males and females, scientists and mathematicians.* Journal of Autism and Developmental Disorders, 2001. **31**(1): p. 5-17.

11. Glans, M., et al., *Self-rated joint hypermobility: the five-part questionnaire evaluated in a Swedish non-clinical adult population.* Bmc Musculoskeletal Disorders, 2020. **21**(1).

12. Allan, N.P., et al., *Identification of anxiety sensitivity classes and clinical cut-scores in a sample of adult smokers: results from a factor mixture model.* J Anxiety Disord, 2014. **28**(7): p. 696-703.

13. Rash, C.J., et al., *Psychometric properties of the IES-R in traumatized substance dependent individuals with and without PTSD.* Addict Behav, 2008. **33**(8): p. 1039-47.

14. Alexander-Bloch, A., J.N. Giedd, and E. Bullmore, *Imaging structural co-variance between human brain regions.* Nat Rev Neurosci, 2013. **14**(5): p. 322-36.

15. Lerch, J.P., et al., *Mapping anatomical correlations across cerebral cortex (MACACC) using cortical thickness from MRI.* Neuroimage, 2006. **31**(3): p. 993-1003.

16. Ma, Z.W. and N.Y. Zhang, *Cross-Population Myelination Covariance of Human Cerebral Cortex.* Human Brain Mapping, 2017. **38**(9): p. 4730-4743.

17. Hänggi, J., D. Wotruba, and L. Jäncke, *Globally altered structural brain network topology in grapheme-color synesthesia.* Journal of Neuroscience, 2011. **31**(15): p. 5816-5828.

18. Raamana, P.R. and S.C. Strother, *Does size matter? The relationship between predictive power of single-subject morphometric networks to spatial scale and edge weight.* Brain Structure & Function, 2020. **225**(8): p. 2475-2493.

19. Epskamp, S., et al., *qgraph: Network Visualizations of Relationships in Psychometric Data.* Journal of Statistical Software, 2012. **48**(4): p. 1 - 18.

20. Friedman, J., T. Hastie, and R. Tibshirani, *Sparse inverse covariance estimation with the graphical lasso.* Biostatistics, 2007. **9**(3): p. 432-441.

21. Kamada, T. and S. Kawai, *An algorithm for drawing general undirected graphs.* Information Processing Letters, 1989. **31**(1): p. 7-15.
